# The immune environment of the mammary gland fluctuates during post-lactational regression and correlates with tumour growth rate

**DOI:** 10.1242/dev.200162

**Published:** 2022-05-03

**Authors:** Jessica Hitchcock, Katherine Hughes, Sara Pensa, Bethan Lloyd-Lewis, Christine J. Watson

**Affiliations:** 1 Department of Pathology, University of Cambridge, Tennis Court Road, Cambridge CB2 1QP, UK; 2 Department of Veterinary Medicine, University of Cambridge, Madingley Road, Cambridge CB3 0ES, UK; 3 Department of Pharmacology, University of Cambridge, Tennis Court Road, Cambridge CB2 1PD, UK; 4 School of Cellular and Molecular Medicine, University of Bristol, University Walk, Bristol BS8 1TD, UK

**Keywords:** Mammary gland, Involution, Immune cells, Tumourigenesis, Mouse

## Abstract

Post-lactational mammary gland regression encompasses extensive programmed cell death and removal of milk-producing epithelial cells, breakdown of extracellular matrix components and redifferentiation of stromal adipocytes. This highly regulated involution process is associated with a transient increased risk of breast cancer in women. Using a syngeneic tumour model, we show that tumour growth is significantly altered depending on the stage of involution at which tumour cells are implanted. Tumour cells injected at day 3 involution grew faster than those in nulliparous mice, whereas tumours initiated at day 6 involution grew significantly slower. These differences in tumour progression correlate with distinct changes in innate immune cells, in particular among F4/80-expressing macrophages and among TCRδ^+^ unconventional T cells. Breast cancer post-pregnancy risk is exacerbated in older first-time mothers and, in our model, initial tumour growth is moderately faster in aged mice compared with young mice. Our results have implications for breast cancer risk and the use of anti-inflammatory therapeutics for postpartum breast cancers.

## Abstract (precis)

**Summary:** Mammary gland involution is associated with dynamic changes in immune cell types and numbers at different stages that correlates with the initial rate of growth of implanted tumour cells.

## INTRODUCTION

The breast undergoes dramatic growth and functional differentiation during pregnancy and lactation and is remodelled to its pre-pregnant state after weaning. This cycle of growth, differentiation and regression is completed with every pregnancy. However, this exposure to continuous fluctuations in hormones and a changing tissue microenvironment has its consequences, and breast cancer is a common disease affecting around 1 in 8 women in the developed world (DeSantis et al., 2014).

Although breast cancer is most common in post-menopausal women, incidence is increasing in women of childbearing age. Interestingly, a full-term pregnancy provides a reduced lifetime risk of breast cancer, but this protective effect is diminished in older mothers, with a first pregnancy after 35 years of age providing no protective effect (Hsieh et al., 1994; Meier-Abt and Bentires-Alj, 2014). Although breastfeeding itself is not associated with increased breast cancer risk, and indeed may provide some protection (Subramani et al., 2021; Subramani and Lakshmanaswamy, 2017), the immediate post-partum period is associated with a 10-30% elevated risk of developing breast cancer (Callihan et al., 2013), and murine studies have also suggested that post-lactational regression is tumour-promoting (Lefrère et al., 2021a). Such postpartum breast cancers (PPBC) are defined by diagnosis during pregnancy or within 5 years of childbirth (Schedin, 2006; Johansson et al., 2011; Macdonald, 2020). Despite being histologically similar to tumours from age-matched non-pregnant women, PPBC tumours are generally of a more advanced stage and are associated with a poorer prognosis (Schedin, 2006; Callihan et al., 2013; Johansson et al., 2011). As the incidence of PPBC increases with age (Lambe et al., 1994), the current trend of delaying childbirth until the fourth decade suggests that PPBC will become more prevalent (Schedin, 2006).

Although their stromal compartments are quite different, the mouse mammary gland (MG) has many similarities to the human breast (McNally and Stein, 2017) with a branched network of ducts embedded within a fatty stroma that contains a variety of immune cell types. In response to pregnancy hormones, extensive tertiary branching and proliferation of milk-producing alveolar cells occurs until, by the day of birth, the fatty tissue is replaced by lobuloalveolar structures that form clusters at the tips of tertiary branches (Watson and Khaled, 2020). During lactation, these lobuloalveoli produce milk, which is expelled into the alveoli lumen by contraction of the basal/myoepithelial cells (Stewart et al., 2021; Masedunskas et al., 2017; Stevenson et al., 2020). When milk is no longer required, the alveolar structures regress and the epithelium is stripped back to the basic ductal architecture (Watson and Khaled, 2020; Watson, 2006b). This post-lactational regression, or involution, is a complex process that couples extensive programmed cell death with tissue remodelling, encompassing redifferentiation of adipocytes in the fatty stroma, and extensive breakdown of extracellular matrix (ECM) components. In mice, involution is associated with extensive changes in gene expression and has a distinct inflammatory signature (Clarkson et al., 2004; Stein et al., 2004).

Postnatal MG development is regulated by immune cells which are dispersed throughout the fatty stroma and closely associate with the ductal network (Wiseman and Werb, 2002; Coussens and Pollard, 2011; Wilson et al., 2022). During pregnancy and lactation, leukocytes, including macrophages, are an integral component of the alveoli, where they intercalate between the basal and luminal epithelial layers, contributing to the regulation of epithelial differentiation (Hitchcock et al., 2020; Dawson et al., 2020; Stewart et al., 2019). During involution, macrophages, eosinophils and mast cells contribute to adipocyte regeneration and re-organisation of the ECM (Gouon-Evans et al., 2002; O'Brien et al., 2010, 2012; Ramirez et al., 2012).

As remodelling of the MG during involution requires the removal of a vast number of dead cells and debris, efficient phagocytosis is essential. This is achieved initially by the mammary epithelial cells (MECs), undergoing a cell fate switch to become phagocytic and subsequently, when these cells die, by an influx of macrophages and other immune cells (Monks et al., 2008; Atabai et al., 2007; Kreuzaler et al., 2011; Fornetti et al., 2016; Hughes et al., 2011, 2012; O'Brien et al., 2012; Sargeant et al., 2014). However, despite the plethora of immune cells and potentially inflammatory stimuli, involution is a non-immunogenic process; pro-inflammatory signals are minimised, preventing tissue scarring and subsequent lactation failure (Wynn, 2008; Sandahl et al., 2010; Teplova et al., 2013; Atabai et al., 2005; Reed and Schwertfeger, 2010).

During lactation and involution, the intra-mammary immune environment becomes tolerogenic to dead cells, debris and milk components (Fornetti et al., 2014; Ballard and Morrow, 2013), adopting a suppressive immune profile consistent with other barrier sites (Betts et al., 2018; Nagy et al., 2021). Immune suppression and tolerance are traits of wound-healing inflammation, which can be associated with tumour promotion and progression (Schäfer and Werner, 2008; Dawson et al., 2020). Indeed, the involuting MG shares several of the hallmarks of cancer (Hanahan and Weinberg, 2011; MacCarthy-Morrogh and Martin, 2020).

The association between PPBC and post-lactational regression largely relies on epidemiological data (Schedin, 2006; Macdonald, 2020) but has been recapitulated in pre-clinical models (Martinson et al., 2015; Lyons et al., 2011; Dawson et al., 2020; Stanford et al., 2014). For example, injection of human tumour cells (MCF10DCIS) into MGs of Severe Combined Immune Deficiency mice 24 h after forced weaning resulted in larger tumours relative to nulliparous controls (Lyons et al., 2011). Although not all studies used immune-competent mice (Lefrère et al., 2021b), several have used targeted immune interventions to directly address the inflammatory component of involution. For example, ablation of Mer tyrosine kinase (MerTK) reduced metastasis to the same level as in nulliparous mice in both spontaneous and allografted mammary tumour models (Stanford et al., 2014); and neutralisation of anti-inflammatory interleukin (IL)10 reduced growth of tumours initiated during involution (Martinson et al., 2015).

The association between the mammary inflammatory profile and tumour growth is becoming more widely acknowledged, yet it remains unclear exactly how involution drives tumour progression. In this study, we carried out a comprehensive analysis of immune cells in MG at different stages of involution and observed striking differences in the first 6 days. We also initiated orthotopic syngeneic tumours at four specific stages using the TUBO tumour cell line, derived from a spontaneously arising tumour in BALB/c-NeuT mice expressing the *Her2/neu* oncogene (Rovero et al., 2000). This is the first time that such a model has been used for an involution study; the commonly used 4T1 or D2A1 cells model the more aggressive triple negative and ductal carcinoma *in situ* (DCIS) breast cancers, respectively. TUBO cells form slower-growing tumours enabling subtle differences in tumour growth to be measured. Interestingly, the different immune cell profiles of the MG at specific stages of involution correlate with corresponding changes in initial tumour growth. Considering the elevated risks of developing PPBC in older first-time mothers, we also compared the rate of tumour growth in cohorts of young and aged mice.

## RESULTS

**Fig. 1.**
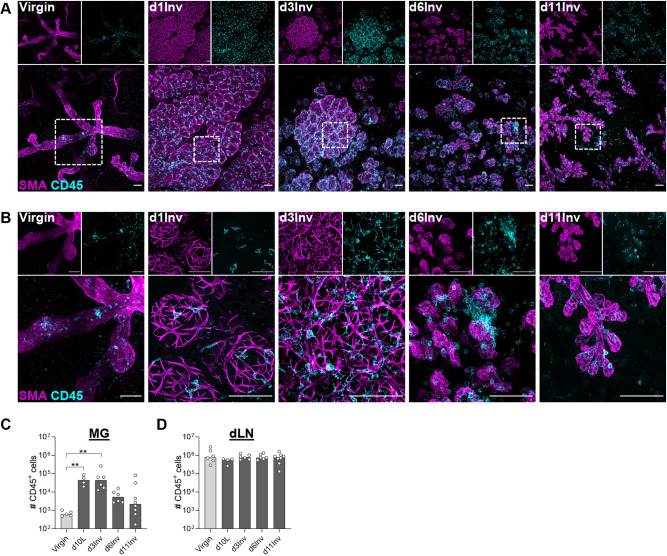
**Optically cleared abdominal MG were imaged in 3D by confocal microscopy in virgin mice and at the indicated time points post-synchronised involution.** (A,B) Maximum intensity projections showing smooth muscle actin-α (SMA)^+^ myoepithelial cells (magenta) and CD45^+^ leukocytes (cyan); white boxed region in A is enlarged in B. Representative images of four mice per group. Scale bars: 100 μm. (C,D) Number of CD45^+^ cells (identified by flow cytometry) in the lower pooled abdominal mammary gland (MG) (C) and the draining (inguinal) lymph node (dLN) (D). C and D include 4-8 mice per group; bars show median. Statistical significance was determined using the Kruskal–Wallis test with Dunn's multiple comparisons test on pre-selected column pairs (**P≤0.01).

Involution is a dynamic process of extensive epithelial cell death and tissue remodelling, requiring an influx of immune cells. The ductal structure in virgin and lactating mice, and throughout an involution time-course, is shown in optically cleared tissue in 3D (Fig. 1A,B), allowing an appreciation of the extensive changes that occur. Notably, the elongated myoepithelial cells, which in virgin glands align with the ducts, adopt a different shape during lactation and early involution, forming basket-like networks over the surface of the luminal alveolar cells. We have shown previously that leukocytes (CD45^+^; also known as PTPRC) intercalate between the ductal epithelial bilayer in virgin MG, whereas during lactation/early involution, these leukocytes co-localise with myoepithelial cells and have a dendritic morphology (Hitchcock et al., 2020). The density of CD45^+^ cells in both the epithelium and stroma is greatest at day 3 involution (d3Inv), decreasing by day 6 involution (d6Inv), and leukocytes associate less with the contracting myoepithelium.

Leukocytes isolated from pooled 4th and 5th MGs were quantified in virgin mice, during lactation and at days 3, 6 and 11 after forced weaning, to ensure a synchronous process (Watson, 2006a). Cell numbers were not normalised to mammary mass, which itself fluctuates during the pregnancy cycle (Knight and Peaker, 1982; Stewart et al., 2016). Therefore, the entire intra-mammary leukocyte population was captured, including cells within the accumulated milk (Atabai et al., 2007; Hitchcock et al., 2020; Nickerson, 1989). Whereas intra-mammary leukocytes increase a median 74-fold by the peak of lactation (d10L), and decrease in number by d6Inv, leukocyte numbers are unchanged in the draining lymph node (dLN) (Fig. 1C,D). Building on previous studies (Jäppinen et al., 2019; Martinson et al., 2015; Betts et al., 2018; Wilson et al., 2020; Dawson et al., 2020), we characterised intra-mammary leukocytes during lactation and involution by flow cytometry to enable phenotypic characterisation of the distinct morphological changes that we observed in the immune milieu (Fig. 1A,B) (Hitchcock et al., 2020).

### Intra-mammary neutrophil frequency peaks at day 6 involution as other myeloid populations are resolving

**Fig. 2.**
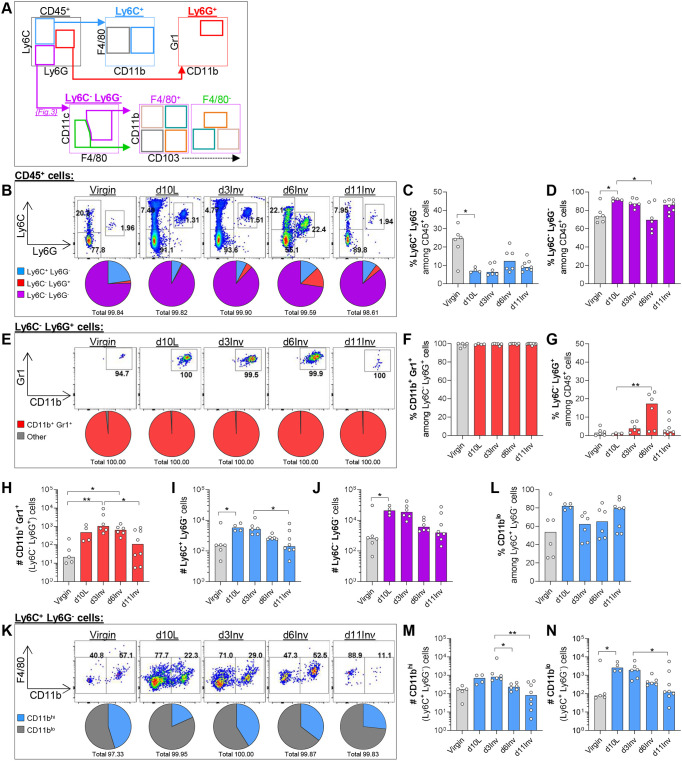
**Leukocytes isolated from pooled abdominal MG were analysed by flow cytometry after forced involution.** (A) Schematic of gating strategy. (B,E,K) Representative dot plots from virgin mice and at the indicated time points post-forced involution; pie charts show frequency of parent population. (C-D,F-J,L-N) Bar charts show frequency of parent population (%) or absolute number of indicated cells from pooled abdominal MGs (referred to as per MG; #), as indicated. All groups include 3-8 mice; statistical significance (Kruskal-Wallis test with Dunn's multiple comparisons test; *P≤0.05, **P≤0.01) was performed on pre-selected pairs. Bar charts show medians.

Innate immune cells were first categorised by Ly6C and Ly6G expression to identify monocytes and neutrophils, respectively, which were subsequently excluded from downstream macrophage characterisation (Fig. 2A). In virgin MGs, 25% of leukocytes express Ly6C, 1% express Ly6G and the remainder are Ly6C^−^Ly6G^−^ (Fig. 2B). During lactation and involution, the proportion of Ly6C^+^ cells decreases with a concomitant increase in the proportion of Ly6C^−^Ly6G^−^ cells (Fig. 2B-D).

Ly6G is expressed by granulocytic leukocytes, which we confirmed were neutrophils using positive CD11b (ITGAM) and Gr1 staining (Fig. 2E,F). Interestingly, the percentage of neutrophils increases during involution, particularly at d6Inv (Fig. 2G). Overall, absolute neutrophil numbers increase by d10L, possibly owing to the increased potential for infection (Stein et al., 2004). Neutrophils remain prevalent at d6Inv whereas numbers of Ly6C^+^ and Ly6C^−^Ly6G^−^ cells are both resolving by this point (Fig. 2H-J).

Ly6C^+^ cells were broadly classified into CD11b^hi^ monocytes and CD11b^lo^ cells in the context of pan-macrophage marker F4/80 (Fig. 2K). Although this gating does not discriminate on F4/80 expression, it enables visualisation of its heterogeneity; monocytes (Ly6C^+^CD11b^+^) can co-express F4/80 to varying degrees (Jäppinen et al., 2019). Here, F4/80 expression was quantified using geometric mean fluorescence intensity (gMFI) (Fig. S1A,B). Despite variability in CD11b expression between mice (Fig. 2L), the absolute number of Ly6C^+^CD11b^hi^ monocytes was fairly constant during lactation/involution, whereas we observed increases in both percentage and number of Ly6C^+^CD11b^lo^ cells (Fig. 2L-N). Considering Ly6C^+^CD11b^lo^ cells generally express lower levels of F4/80, we anticipated this population to include Ly6C^+^ lymphocytes (Hänninen et al., 1997). Among Ly6C^+^CD11b^hi^F4/80^lo^ monocytes, we report elevated C-C-chemokine receptor-2 (CCR2) expression during lactation and involution, which may reflect infiltration from the periphery (Jäppinen et al., 2019). Moreover, low intensity of MHCII and costimulatory activation marker expression further supports the classification of these cells as monocytes, rather than Ly6C-expressing macrophages (Fig. S1C-J).

### Macrophage homeostasis is distinctly altered at day 6 involution

**Fig. 3.**
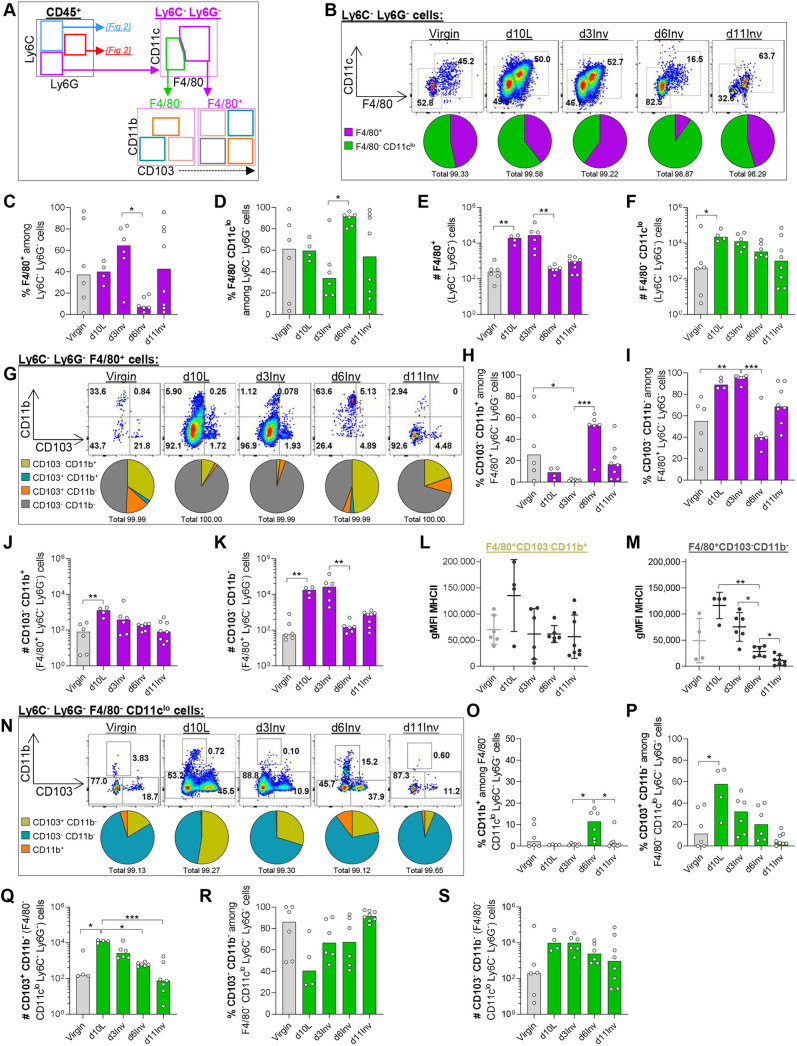
**Leukocytes isolated from pooled abdominal MG were analysed by flow cytometry after forced involution.** (A) Schematic of gating strategy. (B,G,N) Representative dot plots and pie charts of frequencies among parent populations. (C-F,H-K,O-S) Bar charts show frequencies among parent population (%) or absolute cell number per pooled abdominal MGs (#), as indicated. (L,M) Geometric MFI of MHCII expression in the indicated populations of Ly6C^−^Ly6G^−^ cells. All groups include 4-8 mice; statistical significance (bar charts: Kruskal-Wallis non-parametric test with Dunn's multiple comparisons test; dot plots (L,M): one-way ANOVA with Dunnett T3 post-test; *P≤0.05, **P≤0.01, ***P≤0.001) was performed on pre-selected pairs. Bar charts show medians, dot plots show mean±s.d range.

In virgin MGs, 75% (median) of leukocytes are Ly6C^−^Ly6G^−^, comprising macrophages, dendritic cells (DCs) and lymphocytes among other immune cells (Fig. 2D). Macrophages were defined using F4/80 positivity and show varying degrees of CD11c (ITGAX) expression (Fig. 3A), as described previously in MG (Plaks et al., 2015; Dawson et al., 2020). Approximately half of Ly6C^−^Ly6G^−^ cells are F4/80^+^ in virgin MGs, increasing at d3Inv and markedly reducing at d6Inv (Fig. 3B,C). Conversely, the proportion of F4/80^−^CD11c^lo^ cells among Ly6C^−^Ly6G^−^ cells is elevated at d6Inv (Fig. 3D). Absolute numbers of Ly6C^−^Ly6G^−^F4/80^+^ macrophages are increased at d10L and d3Inv, and are fully resolved to virgin levels by d6Inv, whereas non-macrophage Ly6C^−^Ly6G^−^ cells (F4/80^−^CD11c^lo^) remain elevated relative to virgin numbers at d6Inv (Fig. 3E,F).

Macrophages were further characterised using integrins CD11b and CD103 (ITGAE) expression (Fig. 3G). CD103 [recognising E-cadherin (E-cad)] is expressed by multiple leukocyte types including mucosal DCs (Annacker et al., 2005; Sung et al., 2006), intraepithelial lymphocytes (IELs) and regulatory T cells (Uss et al., 2006; Lehmann et al., 2002; Mueller et al., 2013). Here, CD103 expression among F4/80^+^ cells is rare, although we observe distinct CD11b^+^CD103^+^ and CD11b^−^CD103^+^ populations, which are most prevalent at d10L and d3Inv (Fig. 3G; Fig. S2A-D). Back-gating on CD11b^−^CD103^+^ cells revealed that, especially at d10L and d3Inv when the total number of Ly6C^−^Ly6G^−^ cells is greatest (Fig. 2J), CD103^+^ cells have highest CD11c and, generally, lowest F4/80 expression (Fig. S2E). Our gating likely incorporates CD11c^+^F4/80^lo^CD103^+^ DCs described in MG (Wilson et al., 2022) and at other epithelial sites (Ginhoux et al., 2009). However, we also detected rare, previously undescribed F4/80^+^CD11c^+^CD103^+^ cells. We presume that during lactation and involution, a sub-population of F4/80^+^CD11c^+^ cells, ductal macrophages, which closely associate with MECs (Dawson et al., 2020), use CD103 for direct cell-cell signalling. Given their rarity, these CD103^+^ ductal macrophages may be challenging to visualise histologically.

However, CD103 expression among macrophages is exceedingly rare and the majority of F4/80^+^ cells were CD103^−^ (Fig. 3G-I). Although variable in virgin mice, the percentage of CD11b^+^CD103^−^ cells was markedly reduced at d10L and d3Inv and increased at d6Inv (Fig. 3G-I). Total F4/80^+^CD11b^−^CD103^−^ macrophages increased specifically at d10L and d3Inv, whereas F4/80^+^CD11b^+^CD103^−^ macrophage numbers were more constant across all time-points (Fig. 3J,K). Our data support the notion of distinct CD11b^+/−^ mammary macrophage populations, as recently described (ductal CD11c^+^CD11b^lo^ and stromal CD11c^lo^CD11b^+^) (Dawson et al., 2020), which vary in prevalence at different involution stages. We observed increased CD11b^−^ (potentially ductal) macrophages at d10L and d3Inv, although additional markers are required to confirm these stromal/ductal phenotypes (Fig. 3J,K).

Activation was assessed among CD11b^+^CD103^−^ and CD11b^−^CD103^−^ macrophages using MHCII and costimulatory markers CD80/CD86. MHCII intensity was markedly increased among both populations at d10L (Fig. 3L,M; Fig. S2F). Although CD80/86 expression mimics the MHCII expression pattern in CD11b^+^CD103^−^ macrophages, intensity was <10-fold lower in CD11b^−^CD103^−^ macrophages, relative to CD11b^+^CD103^−^ populations, at all time-points (Fig. S2G-J). Reduced costimulatory molecule expression may reflect dampened ability to support/initiate pro-inflammatory signalling. Considering the dominance of CD11b^−^CD103^−^ macrophages at d10L and d3Inv, we may, therefore, expect reduced immune activation at these time points.

Despite their heterogeneity, we consider all F4/80^+^ cells here to be macrophages, although in the absence of additional markers such as CD64 (FCGR1), this is fluid. The differences in surface marker expression among F4/80^+^ cells specifically at d6Inv could infer a change in the inflammatory environment and may include *in situ* macrophage differentiation from infiltrated peripheral monocytes, and/or altered cellular function. Additional markers such as MerTK and CD206 (MRC1) (Wilson et al., 2022) and functional analysis, including metabolic activity (Galván-Peña and O'Neill, 2014), would be required to address this issue.

### Non-myeloid leukocyte dynamics in the involuting MG

Next, Ly6C^−^Ly6G^−^F4/80^−^CD11c^lo^ cells were further characterised using CD11b and CD103 expression. The majority of these cells are CD11b^−^ at all time points, except d6Inv at which we observe a modest increase in the proportion of CD11b^+^ cells (Fig. 3N,O). However, this does not translate into changes in absolute cell number; Ly6C^−^Ly6G^−^F4/80^−^CD11c^lo^CD11b^+^ cells remain low throughout involution, and expression of MHCII and costimulatory markers are modest (Fig. S3A-D).

Although the majority (median 86%) of Ly6C^−^Ly6G^−^F4/80^−^CD11c^lo^ cells are CD11b^−^CD103^−^, CD11b^−^CD103^+^ cells become increasingly prevalent during lactation/involution (Fig. 3P-S). In virgin MGs, 11% of Ly6C^−^Ly6G^−^F4/80^−^CD11c^lo^ cells are CD11b^−^CD103^+^, increasing to >50% at d10L, equivalent to an ∼80-fold increase in absolute number. This resolves during involution (Fig. 3N,P,Q). Intensity of MHCII and CD80/86 are relatively low among Ly6C^−^Ly6G^−^F4/80^−^CD11c^lo^CD11b^−^CD103^+^ cells (Fig. S3E-G), suggesting, along with low/absent CD11c expression and lack of CD11b, that these cells include IELs (discussed below) (Agace et al., 2000).

The remaining Ly6C^−^Ly6G^−^F4/80^−^CD11c^lo^CD11b^−^CD103^−^cells likely also include lymphocytes (CD103^−^ T and B cells), natural killer cells, mast cells, and other immune cells not captured here. Intensity of MHCII and CD80/86 expression among these cells was low, with some increased MHCII intensity at d6Inv and d11Inv (Fig. S3H-J). This may reflect B cell recruitment later during involution, although other reports have identified plasma cell accumulation from d4Inv onwards (Stein et al., 2004).

Overall, we detected multiple changes in myeloid cell homeostasis, which may influence the inflammatory environment at distinct stages during the pregnancy/involution cycle. T cell population dynamics were examined in parallel in the same cohort of mice, using an independent flow cytometry panel. To enable a comprehensive view of the immune landscape in these mice, we examined T cells in both MG and dLN.

### T lymphocyte populations during lactation and involution

**Fig. 4.**
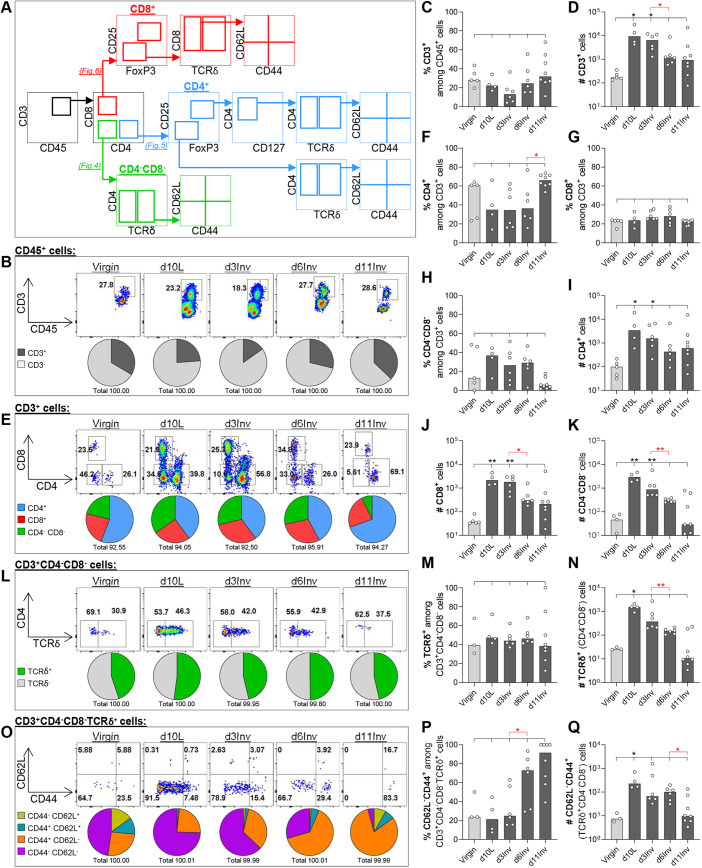
**Leukocytes isolated from pooled abdominal MG were analysed by flow cytometry after forced involution.** (A) Schematic of gating strategy (also used in Figs 5 and 6). (B,E,L,O) Representative dot plots and pie charts of frequencies among parent populations. (C,D,F-K,M,N,P,Q) Bar charts show frequencies among parent populations (%) or absolute number of cells per pooled abdominal MGs (#) as indicated. All groups include 3-8 mice; statistical significance compared with virgin mice (black; Kruskal–Wallis test), additional Mann–Whitney tests (red) between specific pairs indicated (*P≤0.05, **P≤0.01). Bar charts show medians.

T cells were identified using T cell co-receptors CD3, CD4 and CD8 (Fig. 4A). In the dLN, T cell frequency and abundance was unchanged; CD4^+^/CD8^+^ T cell dynamics were constant, and frequency of CD4^−^CD8^−^ cells was negligible (<2% CD3^+^ cells) (Fig. S4A-J). In virgin MGs, ∼30% of leukocytes were CD3^+^ and significantly increased in number at d10L and d3inv (Fig. 4B-D). T cells in virgin MG are predominantly CD4^+^ (60%); the remainder are made up equally of CD8^+^ and CD4^−^CD8^−^ T cells (Fig. 4E-H). During lactation and involution, percentages of CD4^+^, CD8^+^ and CD4^−^CD8^−^ T cells did not change significantly, except for an increased frequency of CD4^+^ cells at day 11 involution (d11Inv), correlating with a reduced percentage of CD4^−^CD8^−^ T cells (Fig. 4E-H). The absolute number of all T cell populations increased significantly at d10L, resolving thereafter, notably with significant reductions between d3-6Inv in CD8^+^ and CD4^−^CD8^−^ cells (Fig. 4I-K).

**Fig. 5.**
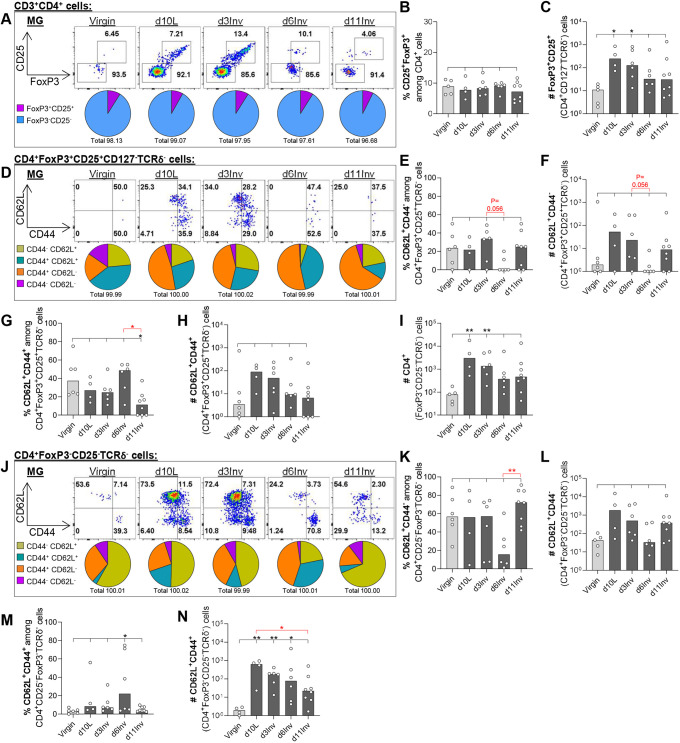
**Leukocytes isolated from pooled abdominal MG were analysed by flow cytometry after forced involution.** (A,D,J) Representative dot plots and pie charts of frequencies among parent populations. (B,C,E-I,K-N) Bar charts show frequencies among parent populations (%) or absolute cell number per pooled abdominal MGs (#), as indicated. All groups include 3-8 mice; statistical significance compared with virgin mice (black; Kruskal–Wallis test), additional Mann–Whitney tests (red) between specific pairs indicated (*P≤0.05, **P≤0.01). Bar charts show medians. Gating strategy used is shown in Fig. 4A.

**Fig. 6.**
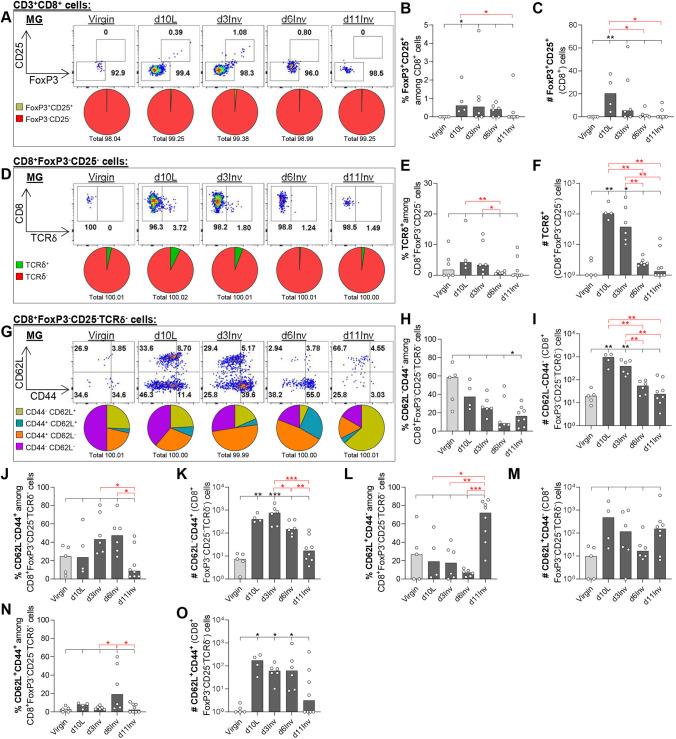
**Leukocytes isolated from pooled abdominal MG were analysed by flow cytometry after forced involution.** (A,D,G) Representative dot plots and pie charts of frequencies among parent populations. (B,C,E,F,H-O) Bar charts show frequencies among parent populations (%) or absolute cell number per pooled abdominal MGs (#), as indicated. All groups include 3-8 mice; statistical significance compared with virgin mice (black; Kruskal–Wallis test), additional Mann–Whitney tests (red) between specific pairs indicated (*P≤0.05, **P≤0.01, ***P≤0.001). Bar charts show medians. Gating strategy used is shown in Fig. 4A.

#### Unconventional T cell subsets

CD3^+^CD4^−^CD8^−^ T cells are associated with the expression of non-classical isoforms of the TCR variable (V) chain (Allison et al., 1991) including gamma-delta (γδ) T cells, which we detected using clone GL3, known to recognise most TCR-Vδ chains (Goodman and Lefrancois, 1989). Approximately half of CD4^−^CD8^−^ T cells express TCRδ at all time points examined in both MG and dLN, corresponding to a significant increase in total γδ T cells in the MG only, throughout lactation and involution (Fig. 4L-N; Fig. S4K-M). Alpha-beta (αβ) T cells can be broadly classified into central memory (CM), effector memory (EM) or naïve cells according to expression of CD44 and CD62L (SELL), among other migration/differentiation markers (Jameson and Masopust, 2009). There is evidence that γδ T cells may share these expression patterns to some degree, such as in lymphoid tissues (Ugur et al., 2018; Zeng et al., 2012), so we used these markers to further phenotype γδ T cells.

Most TCRδ^+^ T cells in dLNs in virgin mice are CD44^−^CD62L^+^ (naïve) whereas CD44^+^CD62L^−^ (recently activated and EM) cells are predominant during lactation and involution (Fig. S4N-P). This may suggest increased γδ T cell trafficking through the dLN during lactation/involution, although the overall frequency of CD4^−^CD8^−^ T cells per dLN is low (Fig. S4J). In MG, many TCRδ^+^ T cells are CD44^−^CD62L^−^, particularly in virgin, lactating and early-involuting MGs (Fig. 4O). The absence of CD62L suggests these cells are unable to home to LNs, and may reflect tissue residency, common among γδ T cells at epithelial sites (Ugur et al., 2018; Hayday and Tigelaar, 2003). The percentage of CD44^+^CD62L^−^ [recently activated, EM and tissue resident memory (TRM)] γδ T cells increases significantly at d6Inv, although total numbers are unchanged between d3Inv and d6Inv (Fig. 4O-Q) (Schenkel and Masopust, 2014). Frequencies of memory cell classifications among TCRδ^−^ CD4^−^CD8^−^ T cells, which may represent additional non-GL3-associated TCR-Vδ chains, are shown (Fig. S5A,B). Together, these data quantitatively describe potentially activated γδ T cells in both MG and dLN, which increase in frequency and number during involution.

#### Conventional CD4^+^ T cells

Regulatory cells were identified among conventional CD4^+^ cells using transcription factor FoxP3, IL2 receptor α-chain (CD25; IL2RA), and CD127 (IL7R) expression (Fig. 4A). The proportion of CD4^+^FoxP3^+^CD25^+^ cells (median 7-9%, of which ∼100% are CD127^−^ and TCRδ^−^) is unchanged during lactation and involution (Fig. 5A,B; Fig. S6A-D). Despite this, the absolute number of CD4^+^ Tregs, which was extremely low in virgin MGs, increased a median 23-fold at lactation (Fig. 5C). Memory and activation of Tregs were characterised using CD62L and CD44. The most striking observation was the increased frequency of CD62L^+^CD44^+^ (CM) and reduced frequency of CD62L^+^CD44^−^ (naïve) Tregs at d6Inv, which translates to changes in absolute numbers (Fig. 5D-H; Fig. S6E-H). These changes, particularly loss of naïve Tregs at d6Inv, were specific to MG and are not observed in the dLN (Fig. S7A-K).

In the MG, the remaining (∼90%) CD4^+^ T cells were FoxP3^−^CD25^−^ effector T helper cells, which significantly increased in number at d10L/d3Inv (Fig. 5A,I). Incidentally, among CD4^+^FoxP3^−^CD25^−^ cells, rare TCRδ^+^ cells were even less frequent at d10L, d3Inv and d6Inv relative to virgin and d11Inv (Fig. S6I,J). CD62L/CD44 expression in CD4^+^FoxP3^−^CD25^−^TCRδ^−^ cells, although variable between mice, showed a reduction in naïve (CD62L^+^CD44^−^) cells specifically at d6Inv (Fig. 5J-L). Also, we observed a significant increase in CD62L^+^CD44^+^ (CM) numbers throughout lactation and involution, although these cells constitute a minor percentage of total effector T helper cells in MG (Fig. 5J,M,N; Fig. S6K-N). These changes were not reflected in dLN, where numbers and proportions of all CD4^+^ populations examined were largely unchanged (Fig. S7L-R).

#### Unconventional and conventional CD8^+^ T cells

Regulatory CD8^+^ T cells (CD8^+^FoxP3^+^CD25^+^) were very low in frequency in MG across all time points (<1% total CD3^+^CD8^+^ cells), although the absolute number increased significantly and specifically at d10L (Fig. 6A-C). Moreover, among these rare cells, an increased proportion expressed TCRδ at d3Inv (Fig. S8A). Considering the highly immune-suppressive nature of CD8^+^ γδ T cells in diabetes (Harrison et al., 1996), the presence of these rare cells is worth noting. TCRδ expression was examined among the remaining CD8^+^FoxP3^−^CD25^−^ cells to determine whether CD8^+^ IELs (non-conventional T cells reported in other epithelial effector sites) may be detected in the involuting MG (Hedrick et al., 1985). CD8^+^FoxP3^−^CD25^−^TCRδ^+^ cells increased modestly in frequency at d10L and d3inv, corresponding to a significant increase in absolute number at these time points (Fig. 6D-F).

Lastly, we examined activation/memory among conventional non-regulatory CD8^+^TCRδ^−^ T cells. As with CD4^−^CD8^−^ cells (but not CD4^+^ T cells), a substantial proportion of CD8^+^FoxP3^−^CD25^−^TCRδ^−^ cells were CD44^−^CD62L^−^ in virgin MGs, which may indicate tissue residency (Fig. 6G,H). The absolute number of CD8^+^FoxP3^−^CD25^−^TCRδ^−^CD44^−^CD62L^−^ cells increased significantly at d10L and d3Inv, before significantly falling at d6Inv (Fig. 6I). Moreover, activated CD8^+^FoxP3^−^CD25^−^TCRδ^−^ cells also increased/decreased in number significantly at these same time-points, respectively, indicating a broad switch in CD8^+^ T cell dynamics between virgin and d10L, and between d3Inv and d6Inv (Fig. 6H-K). Interestingly, the percentage of CD44^−^CD62L^+^ (naïve) CD8^+^FoxP3^−^CD25^−^TCRδ^−^ cells was increased specifically at d11Inv, maintaining cell numbers at this time point (Fig. 6L,M). CM (CD62L^+^CD44^+^) CD8^+^FoxP3^−^CD25^−^TCRδ^−^ cell numbers were significantly elevated between d10L and d6Inv, relative to virgin MGs (Fig. 6N,O). Despite these considerable changes in CD8^+^ T cells in MG, these cell populations were largely unchanged in the dLN, with the exception of a reduction in CD8^+^ regulatory cells between d3Inv and d6Inv (Fig. S8B-L).

Overall, altered T cell homeostasis, particularly among regulatory and unconventional T cells, may contribute to altered tumourigenic potential in the MG during involution.

### Tumour growth rate depends on the involution stage at which tumour cells are implanted

**Fig. 7.**
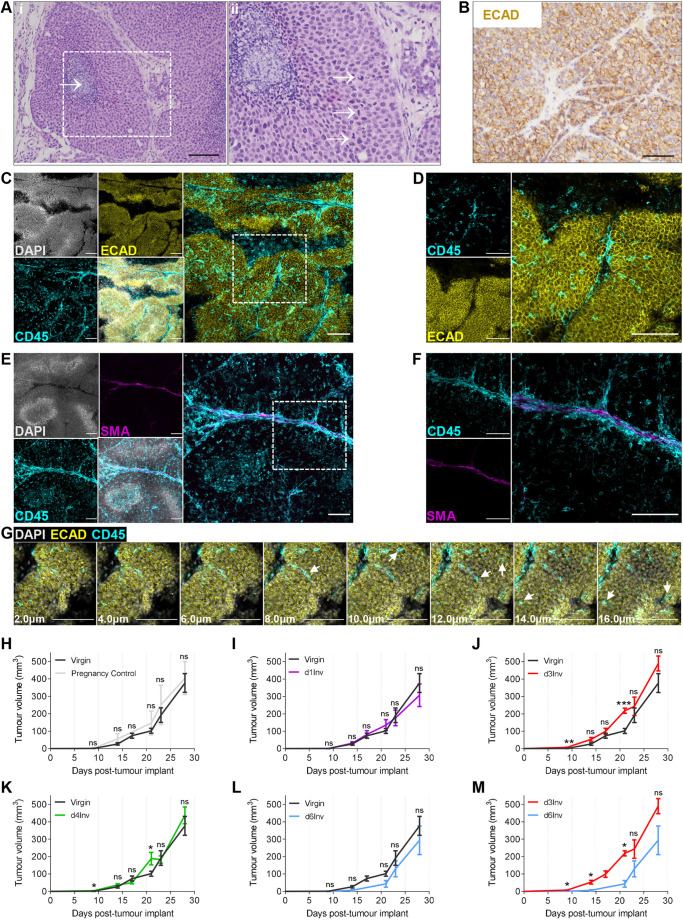
**TUBO cells were implanted into the abdominal MG and tumours were harvested after 26 days.** (A,B) 2D sections showing H&E (A) and E-cadherin (ECAD) (brown; Haematoxylin counterstain) (B). The boxed region in Ai is enlarged in Aii. White arrow in Ai shows central necrotic foci; white arrows in Aii show bizarre mitoses. (C-G) Maximum intensity projections (MIPs) of optically cleared TUBO tumours imaged in 3D by confocal microscopy. (C,D) ECAD (yellow), CD45 (cyan), DAPI (grey); merged in large panels; boxed region in C is enlarged in D. (E,F) Smooth muscle actin-α (SMA) (magenta), DAPI (grey), CD45 (cyan); merged in larger panels; boxed region in E is enlarged in F. (G) MIPs of sequential optical slices 1 μm thick; the depth from the first image in the sequence is provided; arrows show CD45^+^ leukocytes (cyan) only visible within the lobule. Scale bars: 80 μm (A); 40 μm (B); 100 μm (C-G). All images are representative of >4 mice per group. (H-M) TUBO cells were implanted into the abdominal MG of virgin or pregnancy control mice, or at the indicated times after forced involution (data from J and L are combined in M); mean tumour volume shown; groups contained 3-9 mice. Statistical significance: Mann–Whitney tests were performed at individual time points (*P≤0.05, **P≤0.01, ***P≤0.001). ns, non-significant. Tumour growth presented as mean±s.e.m.

Accelerated tumour growth has been reported in mammary tumours initiated at day 1 involution (d1Inv) relative to virgin mice (Martinson et al., 2015). Considering the contrasting immune repertoire we observed at distinct stages of involution, particularly between d3Inv and d6Inv, we sought to address whether these immune environments correspond to different initial tumour growth rates. Orthotopic implantation of TUBO cells (Rovero et al., 2000) into mammary fat pads resulted in tumours with multifocally prominent lobules, sometimes with central necrotic foci (Fig. 7Ai). Evidence of mitotic activity was abundant; bizarre mitoses were observed, inferring high levels of proliferation (Fig. 7Aii). TUBO cells expressed E-cad on the plasma membrane (Fig. 7B) and 3D imaging revealed distinct E-cad^+^ lobules segmented by α-SMA^+^ stroma, likely consisting of vasculature and fibroblasts and other cell types (Fig. 7C,E). TUBO tumours were moderately infiltrated by CD45^+^ leukocytes, which accumulated in the α-SMA^+^ inter-lobular regions and were dispersed throughout the lobules, as visualised through individual *z*-sections (Fig. 7D,F,G).

Tumour growth rate (by volume) was comparable between virgin and pregnancy control mice (see Materials and Methods for definitions), indicating that pregnancy-associated factors have little influence on tumour growth, although inter-mouse variability was high (Fig. 7H; Fig. S9A,B). We therefore consider the TUBO model suitable to study the role of involution-associated factors in tumour progression, using virgin females alone as controls.

To determine whether different involution stages correspond to altered tumour growth, TUBO cells were orthotopically implanted into surgically exposed MGs (Tavera-Mendoza and Brown, 2017) at days 1, 3, 4 and 6 after synchronised involution. Interestingly, implantation at d1Inv resulted in almost identical tumour growth kinetics to virgin mice, whereas tumours implanted at d3Inv-d4Inv grew moderately faster, and those implanted at d6Inv grew moderately slower (Fig. 7I-L; Fig. S9A-F). Thus, tumour growth rate is significantly different at d3Inv compared with d6Inv, suggesting the microenvironment at these times differentially promotes tumour growth (Fig. 7M).

### Elimination of anaesthesia/analgesics from tumour implant procedure

In these studies, TUBO cells were injected into surgically exposed MGs under anaesthesia (Tavera-Mendoza and Brown, 2017). As systemic analgesics could influence the inflammatory microenvironment of the MG, we also implanted TUBO cells by direct injection into the MG without surgery or sedation. Using this less invasive procedure, the injection site was equivalent and tumours developed within the mammary fat pad adjacent to the inguinal lymph node (Fig. S9G).

**Fig. 8.**
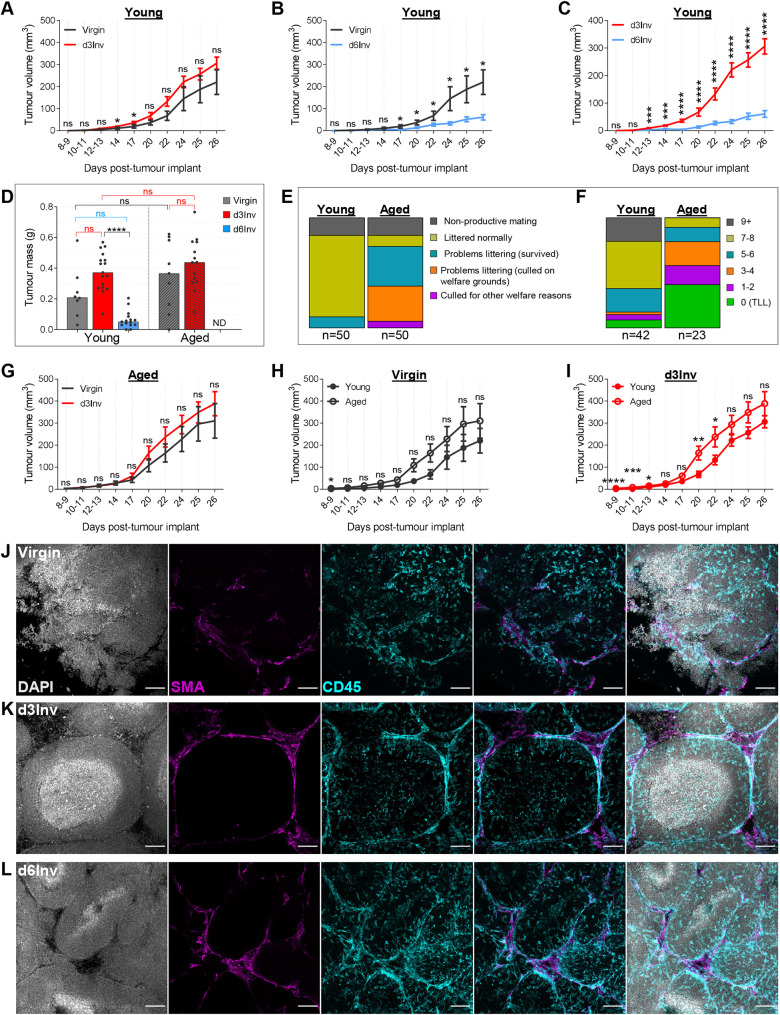
**Young (6-8 week) and aged (10 month) BALB/c mice were mated and TUBO cells were implanted (non-invasively) into the abdominal MG of virgin, d3Inv or d6Inv mice.** (A-C) Mean tumour volume in young mice at the indicated time points; (data in C is the same as that shown for d3Inv and d6Inv in A and B, respectively). (D) Tumour mass at 26 days post-implant in young and aged mice. (E) Breeding outcome of young versus aged mice (50 mice per group); non-productive mating is a visually confirmed pregnancy which failed. (F) Pups per litter of successful pregnancies; groups absent from chart reflects zero mice in group. (G) Mean tumour volume in aged mice. (H,I) Tumour volumes in A and G were replotted for comparison of young versus aged mice (experiments were performed in parallel). (J-L) Optically cleared TUBO tumours from young mice harvested after 26 days, imaged in 3D by confocal microscopy; DAPI (grey), SMA-α (magenta), CD45 (cyan); right panel shows merged image. Scale bars: 100 μm. Groups=8-17 mice. Statistical significance: Mann–Whitney tests performed at individual time-points (*P≤0.05, **P≤0.01, ***P≤0.001, ****P≤0.0001). ND, not determined; ns, non-significant; TLL, total litter lost. Bar charts show medians; tumour growth presented as mean±s.e.m.

Without the use of anaesthesia or analgesics, tumours implanted at d3Inv grew moderately faster, whereas those implanted at d6Inv grew significantly slower relative to virgin mice (Fig. 8A,B; Fig. S10A-C). Moreover, direct comparison between implantation at d3Inv and d6Inv revealed significantly altered tumour outgrowth (Fig. 8C,D), with the difference being accentuated in the absence of non-steroidal anti-inflammatory agents (Caprofen and Meloxicam). This striking difference suggests that the inflammatory environment of the MG can influence initial tumour outgrowth rate, although this has not been addressed experimentally here.

### Tumour growth is moderately enhanced in aged mice

Human epidemiological evidence suggests that in older first-time mothers (35+ years), pregnancy increases breast cancer risk (Schedin, 2006). To examine the extent to which the involution microenvironment affects tumour outgrowth in aged first-time dams, a cohort of virgin mice were aged for 10 months, equivalent to approximately 38 human years.

Pregnancy and littering was more challenging in the aged cohort, with a considerably reduced proportion of dams able to produce a viable litter of >5 pups (Fig. 8E,F). Furthermore, unexpectedly, specifically at d3Inv, nearly all aged dams were clinically exhausted, showing piloerection and significantly reduced activity. This was not observed in young mice or in aged multiparous mice nor was it encountered in our preliminary studies on aged mice where TUBO cells were implanted at d1Inv in anaesthetised animals receiving post-operative analgesics for 24-48 h. We presume the sedation/analgesics masked clinical symptoms at d3Inv. Importantly, all aged dams which produced viable litters or accepted foster pups, nurtured their young, indicating no lactation abnormalities.

Due to these considerable difficulties, we could only examine tumour outgrowth at one involution time point. TUBO cells implanted into the MGs of 10-month-old mice at d3Inv grew moderately faster than those in virgin mice, although this did not reach statistical significance (Fig. 8D,G; Fig. S10D,E). Direct comparison between young and aged mice revealed a tendency for aged mice to have faster growing tumours in both groups, which reached statistical significance in the d3Inv group, especially at the earliest stages of tumour outgrowth (Fig. 8H,I; magnified in Fig. S10F,G). Importantly, tumours from all groups were morphologically similar, as shown by 3D imaging (shown for the young cohort only) (Fig. 8J-L) and by Haematoxylin and Eosin (H&E) for all groups (Fig. S10H).

Taken together, our data show that tumourigenicity differs throughout involution and that this coincides with changes in immune cell types and frequencies. These findings further our understanding of tumour growth in the context of post-lactational regression, whereby we observe differences in the immune landscape between d3Inv and d6Inv that could contribute to the altered tumour outgrowth observed.

## DISCUSSION

Mammary involution in immune-competent mice provides an excellent experimental system to examine tumour growth in a physiologically immune-suppressive state. Post-pregnancy, the entire tissue is regenerated in a highly regulated process, enabling developmental changes to occur while avoiding widespread inflammatory activation of the immune system (Atabai et al., 2007; Sandahl et al., 2010).

### Altered innate immune homeostasis correlates with tumour growth rate

Intra-mammary immune cells have been previously characterised at various developmental stages using flow cytometry and imaging (Martinson et al., 2015; Betts et al., 2018; Hitchcock et al., 2020; Dawson et al., 2020; Plaks et al., 2015; Stewart et al., 2019; Jäppinen et al., 2019). Here, we build a comprehensive understanding of this immune environment by coupling immune cell characterisation with rate of tumour outgrowth at distinct points throughout involution. We show that tumours implanted at d3Inv, when involution is characterised by extensive cell death and alveolar collapse, grow faster than those implanted into virgin mice. Moreover, and perhaps more surprisingly, we demonstrate that tumour growth is significantly reduced when tumours are initiated at d6Inv. These differences in tumour progression correlate with distinct changes in immune cells, particularly among F4/80^+^ macrophages and TCRδ^+^ unconventional T cells.

### Macrophage phenotype correlates with tumour growth at d3Inv-d6Inv

Using flow cytometry, we observe a marked reduction in the proportion of F4/80^+^ macrophages at d6Inv, and absolute numbers fall to those observed in virgin mice (Fig. 3B,C,E). At this time, collapsed alveoli are regressing as luminal alveolar MECs die, myoepithelial MECs contract (Hitchcock et al., 2020) and total leukocyte numbers are diminishing as the tissue returns to its pre-pregnant state.

Of the F4/80^+^ macrophages remaining at d6Inv, we observe an increased percentage of CD11b^+^CD103^−^ cells (Fig. 3G,H). CD11b has been shown to influence myeloid cell polarisation by promoting inflammatory gene expression in macrophages (Schmid et al., 2018). Moreover, absence of CD11b has been associated with enhanced tumour growth both in CD11b-deficient mice and using adoptive transfer of CD11b-null macrophages (Schmid et al., 2018). It is intriguing, therefore, that the trend for reduced CD11b expression among F4/80^+^ cells observed at d10L and d3Inv is not seen at d6Inv. This may indicate that macrophages at d6Inv have more inflammatory capacity than those at d10L and d3Inv, which may be comparatively more immune suppressed. Whether these changes reflect distinct macrophage population changes, as recently described (Dawson et al., 2020; Wilson et al., 2022), or fluctuations in CD11b expression indicative of differences in myeloid cell maturation at these distinct periods of mammary regression and regeneration remains to be seen.

### CD103-expressing cells in the involuting MG

Among F4/80^+^ macrophages, we describe two rare CD103-expressing populations: CD11b^+^CD103^+^ and CD11b^−^CD103^+^, the latter of which is the more prevalent, peaking in number at d10L and d3Inv (Fig. 3G; Fig. S2A-E). Back-gating revealed that CD11b^−^CD103^+^ cells tend to be among the highest CD11c- and lowest F4/80-expressing cells and so may include CD103^+^ DCs described elsewhere (Ginhoux et al., 2009; Agace et al., 2000). However, among these CD103^+^ cells, some clearly express F4/80 in both CD11b^+^/^−^ populations (Fig. S2E). Presumably these cells, possibly sub-populations among F4/80^+^CD11c^+^ ductal macrophages (Dawson et al., 2020), use CD103 for direct cell-cell signalling with MECs during lactation and early involution. Moreover, MHCII and costimulatory marker expression in rare CD11b^+^CD103^+^ macrophages, although variable, is very high, further inferring the functional importance of these cells; CD11b^−^CD103^+^ cells have a much lower intensity of these markers (Fig. S2K-P). We can speculate that any CD103/E-cad-dependent interaction between macrophages and MECs may enable modulation of MEC function via an outside-in signalling axis (Agace et al., 2000).

Concomitantly, CD103 expression is observed among Ly6C^−^Ly6G^−^F4/80^−^CD11c^lo^CD11b^−^ cells, which we presume to be IELs (Agace et al., 2000; Mayassi and Jabri, 2018). These cells increase ∼80-fold in number by d10L and diminish during involution (Fig. 3N,P,Q). CD103 expression is rare on peripheral and splenic T cells, but is widely expressed by T cells (mainly CD8^+^) at mucosal sites such as the gut (Kilshaw and Murant, 1990). Moreover, CD103 expression can be induced by transforming growth factor-1, which is upregulated in MG during involution (Clarkson et al., 2004). Whether the mucosal-like immunity, described previously in the involuting MG (Betts et al., 2018), can be attributed to CD103^+^ IELs remains to be fully elucidated, but our data would support this notion.

### Unconventional T cell dynamics correlate with altered tumour growth

Unconventional T cells, including TCRγδ cells, can be viewed as an intermediary between innate and adaptive immunity, because they can become activated without direct TCR stimulation, enabling them to respond quickly with effector function to inflammatory stimuli (Strid et al., 2008). Although γδ T cells have been described before during lactation, little is known of their role in MG development and pregnancy (Reardon et al., 1990; Fehrenkamp and Miller, 2019). We demonstrate accumulation of CD4^−^CD8^−^TCRδ^+^ T cells in MG during lactation/involution, and observe increased activation (CD62L^−^CD44^hi^) of these cells specifically at late involution time points (d6Inv and d11Inv). Unconventional T cells are known to respond to damage-induced self-antigens ‘trauma signal surveillance’ at epithelial sites (Allison et al., 1991), therefore their increased presence during involution could reflect surveillance of damaged cells and/or cytokine release, which are elevated during involution.

In terms of anti-tumour immunity, there is increasing evidence of immunosurveillance by γδ T cells in epithelial tissues. In one pivotal study, these cells were classed as the most favourable intra-tumoural immune cell type in terms of their prognostic association (Girardi et al., 2001; Gentles et al., 2015). The increased activation among TCRδ^+^ cells we observe at d6Inv and d11Inv may indicate enhanced anti-tumour activity at these points and correlates with the slower growth in tumours initiated at d6Inv. On the other hand, we detect increased CD8^+^TCRδ^+^ cells specifically at d10L and d3Inv. These cells have been associated with immune suppression in some situations, which would correlate with the accelerated tumour outgrowth observed at d3Inv (Harrison et al., 1996). However, until further studies using adoptive cell transfer and cell depletion are performed, the function of the unconventional T cells described here is speculative.

### TUBO cells: a model for immune association of pregnancy associated breast cancer

TUBO cells offer a slow-growing syngeneic tumour model, which has not been used previously in the context of mammary involution. Orthotopic implantation of a low number of cells results in steady tumour progression, which can be tracked over multiple weeks. Although we observed differences in tumour outgrowth, which correlate with differences in immune cells within the MG, particularly at early times after tumour initiation, we found these growth differences to be accentuated in mice that were not administered sedation/post-operative anti-inflammatory analgesics. This supports the notion that the inflammatory environment of the gland at the time of tumour cell implantation affects initial tumour outgrowth.

The interplay between inflammation and tumourigenesis is widely accepted, with extensive literature on use of anti-inflammatory agents in both cancer prevention and management (Wong, 2019; Zhao et al., 2017). However, controlled studies are required to determine the extent to which these agents influence the inflammatory environment during mammary involution, whether they affect tumour outgrowth and, indeed, whether they may be useful therapeutically in this setting. COX-2 (PTGS2) inhibition has been addressed during mammary involution, demonstrating reduced lung metastasis due to disruption of lymphatic dissemination of tumour cells (Lyons et al., 2014). Indeed, inflammation is a multifaceted phenomenon not restricted to immune cells; lymphatic endothelial cells (Elder et al., 2018), fibroblasts (Valdés-Mora et al., 2021), adipocytes (Zwick et al., 2018) and a plethora of other stromal cells and components (Lyons et al., 2011) contribute to the mammary microenvironment, potentially influencing tumour outgrowth during involution. Moreover, the influence exerted by these different cell types may vary depending on the involution stage. For example, mammary adipocytes undergo cyclic remodelling during the pregnancy cycle, with contrasting biological processes occurring at distinct involution stages (Zwick et al., 2018).

In addition to stromal factors, the MECs themselves can also affect tumourigenicity. For example, tumour associated macrophages (TAMs) can be programmed by tumour epithelial cells to promote a favourable immune environment for tumour growth (Dawson et al., 2020; Franklin et al., 2014). Ductal macrophages can share a similar genetic signature to TAMs, and therefore may be educated by MECs (Dawson et al., 2020). Signal transducer and activator of transcription 3 (Stat3), a key orchestrator of immune suppression, becomes highly activated in MECs at the onset of involution (Kritikou et al., 2003; Tiffen et al., 2008). This induces macrophage upregulation of arginase 1 and chitinase 3-like 1, contributing to the anti-inflammatory environment (Hughes et al., 2012). Notably, Stat3 is constitutively active in invasive breast cancers (Watson and Miller, 1995) and, when expressed in tumour epithelial cells, is crucially important in promoting an immunosuppressive microenvironment both during tumour initiation and progression (Jones et al., 2016).

### The influence of age

Since age at first pregnancy affects breast cancer risk, we also used our model to examine whether the effect of involution is modified by the age of the dam, the first time this issue has been addressed using animal models. Here, we report that TUBO tumours tend to grow more aggressively in aged mice. This was unexpected because, although cancer incidence increases with age, tumours are generally more aggressive in younger individuals, both in mice and humans (Fane and Weeraratna, 2020). However, the ‘aged’ mice used here approximate to 38 human years, thus we are not examining tumour growth under severe immune senescence (Goronzy and Weyand, 2013). With this in mind, in future studies it will be vital to understand how immune cells differ in aged versus young MGs before tumour implantation, and whether this accentuates the effect of the involution microenvironment on tumour growth.

The unexpected clinical collapse encountered in aged dams at d3Inv is congruent with a hyper-inflamed phenotype, with the symptoms presumably masked by analgesics. Clinical symptoms prevented the further examination of tumour cell implantation at d6Inv in the aged cohort. Nevertheless, although these studies are incomplete, our observations will inform future studies that will be important to address the increased incidence of PPBC in older first-time mothers.

### Conclusion

Although we, and others, have characterised intra-mammary immune cells at different stages of involution, and we have now correlated this with altered tumour outgrowth, we did not address whether the immune component of the tumours themselves differs in tumours initiated at different involution stages, as we sought to address only whether the inflammatory context of the MG altered initial tumour growth. It is well established that tumours modulate their own microenvironment and so analysis of immune cells at the experimental end point, although interesting, would be conflated by the reciprocal interactions between the tumour and its microenvironment.

Here, we show altered tumourigenicity at distinct stages of post-pregnancy mammary involution, which correlates with intra-mammary immune cells at the time of cell implantation. It is unclear why such striking differences in tumour growth are observed between d3Inv and d6Inv, and not between other time points, which also elicit significant changes in immune composition. However, when tumours were initiated non-invasively, they tended to be slower-growing in the pregnancy control group than in the virgin and non-productive mating groups (Fig. S10I-K). This may suggest that pregnancy in some way influences tumourigenic potential of the gland. Possibly, the developmental changes that occur only in the ductal structures may be important (Wilson et al., 2022), although this is speculative and our data were not statistically significant.

Whether these observations translate to human breast cancer is as yet unknown. However, evidence suggests that the stromal microenvironment has an important role in the progression of early-stage breast cancer (Bussard et al., 2016). The transition from lactation to post-weaning breast involution has not been rigorously evaluated in healthy women, but histological evidence in mice mirrors that in human breast, supporting the use of preclinical models in this field (Jindal et al., 2020).

## MATERIALS AND METHODS

### Mice

Female virgin BALB/c mice were purchased from Charles River Laboratories and were housed thereafter at the Biological Services Unit (BSU), Department of Pathology, University of Cambridge, UK. Mice were purchased at 6-7 weeks old and acclimatised for 7 days in-house before experimental use. Young female mice were set up in breeding pairs/trios at 7-9 weeks of age. Aged females were housed at Charles River Laboratories for 10 months before transfer to our BSU. Male mice (either BALB/c or C57BL6/J) were purchased from Charles River Laboratories; proven studs were used repeatedly for <6 months before being replaced. All animals were housed in a 12 h light/12 h dark cycle facility in individually ventilated cages with access to food and water at all times.

All experiments were performed under licence according to the Animals (Scientific Procedures) Act 1986 and the European Union Directive 86/609 and were approved by the appropriate local animal ethics committee of the University of Cambridge.

### TUBO cells

Tumour studies used the TUBO syngeneic cloned cell line, originally established from a spontaneous mammary carcinoma in a BALB-neuT mouse (Rovero et al., 2000) and were a kind gift from Professor Valeria Poli, University of Turin, Italy. TUBO cells were maintained in DMEM (Gibco, Life Technologies) supplemented with 10% foetal calf serum (Sigma-Aldrich) at 37°C in a humidified atmosphere of 5% CO_2_. Media was replenished every 2-3 days and cells were routinely tested for mycoplasma. Cells were harvested using Trypsin (Sigma-Aldrich) and were quantified by Trypan Blue exclusion.

### Orthotopic tumour implantation

We implanted 5×10^3^ TUBO cells (20 μl injection volume in PBS) orthotopically into the abdominal MG. For implantation under sedation, Isofluorane (induction at 4 l/min, maintenance at 2-2.5 l/min) was used in conjunction with the analgesics carprofen [Rimadyl; 50 mg/ml, dose dependent on weight, administered subcutaneously (s.c.) 30 min before pre-surgery preparation] and buprenorphine (Temgesic or Vetergesic; 0.3 mg/ml, dose dependent on weight, administered s.c. immediately before surgery). Cells were injected into the exposed MG via a <6-7 mm cutaneous incision, as described previously (Tavera-Mendoza and Brown, 2017; Zhang et al., 2018). The incision wound was sealed using surgery clips, removed ∼48 h post-surgery. Mice were housed in a pre-warmed cage (28°C) for ∼30 min post-surgery and remained in surgical bedding for <7 days. Mice were closely monitored <48 h post-surgery and were provided with Metacam (2-4 mg/kg orally) at the vet's discretion. For implantation without sedation, TUBO cells (as above) were injected s.c. into the abdominal MG; no analgesic or other pain relief was administered at any time. Tumour growth in all animals was monitored from day 8 post-implantation using callipers; tumour volume=(4π/3)×(width/2)2×(length/2).

### Involution experiments

Virgin female mice were caged in groups of 5-8 (determined by weight), in accordance with Home Office regulation. Females were re-housed into fresh cages in pairs 1 day before the stud male was introduced. Males were removed after 2 weeks to ensure littering females were not subsequently re-impregnated. When pregnancy was visually confirmed, females were re-housed individually in fresh cages for accurate monitoring of littering onset.

Females which were not pregnant after 2 weeks co-housed with a proven stud were co-housed with other ‘non-pregnant’ females; (included females which did not become pregnant, and females which may have lost the pregnancy in the early stages, before visual confirmation of pregnancy). Litters were normalised (<2 days of birth) to 6-8 pups per dam. For the ‘pregnancy control’ group, all pups were removed immediately at birth (before 08:00 for mice which littered during the night). Dams with pups were left to lactate naturally for 10 days (range 9-11); all pups were removed synchronously (force involution). Dams were re-housed with <5 females of a similar age, in fresh cages.

For tumour implantation into involuting mice, TUBO cells were implanted (as above) at the specified time after the removal of pups. For immune cell characterisation, dams were instead killed at the specified time and tissues excised for *ex vivo* analysis. For lactation time-points [10 days (range 9-11) after littering], TUBO cells were implanted (as above) and dams placed in a fresh cage, or the dam was killed for *ex vivo* analysis.

Control virgin mice (never housed with a male) or control non-pregnant mice (exposed to a male for 2 weeks but did not become visually pregnant) were not oestrus staged but were aged matched; extra mice were purchased and were co-housed alongside the mice described above for these purposes, and thus were subject to all the same environmental conditions as the experimental mice.

### Flow cytometry

For *ex vivo* characterisation of immune cells, the abdominal MGs on one side only were pooled for each mouse. MGs were mechanically disrupted and submerged in PBS containing collagenase A (10 mg/ml; Roche), hyaluronidase (1000 U/ml; Sigma-Aldrich) and DNAse1 (5 µg/ml; Sigma-Aldrich) for 25 min at 37°C with gentle agitation; reactions were stopped with 0.5 mM EDTA. MGs were homogenized through a 70 μm nylon cell strainer (BD Falcon) in RPMI +2% foetal bovine serum +5 mM EDTA. Leukocytes were enriched for using centrifugation with Ficoll Paque PLUS (GE Healthcare) at 350 ***g*** for 20 min without brake. Intra-mammary leukocytes were collected from the interface layer. dLNs were enzymatically digested (as above) but were not subject to density gradient centrifugation. Cells were quantified using Trypan blue exclusion and were stained for flow cytometric analysis using the antibodies listed in Table S1, in conjunction with LIVE/DEAD fixable stain (Invitrogen). For staining of intracellular targets, samples were fixed and permeabilised using commercially available reagents (eBioscience). All samples were acquired using an LSR Fortessa (BD Biosciences) in conjunction with FACSDiva Software (BD Biosciences) at the National Institute for Health and Care Research (NIHR) Cambridge BRC Cell Phenotyping Hub, Department of Medicine, University of Cambridge, UK. Data were analysed using FlowJo (BD Biosciences).

### Optical tissue clearing and three-dimensional immunohistochemistry

Tissues were optically cleared using a modified version of the CUBIC procedure, as previously described (Lloyd-Lewis et al., 2016; Susaki et al., 2014). The following buffers were used; CUBIC reagent 1A {urea [10% (w/w)], *N*,*N*,*N*′,*N*′-tetrakis(2-hydroxypropyl)ethylenediamine [5% (w/w)], Triton X-100 [10% (w/w)] and NaCl (25 mM) in distilled water}; CUBIC reagent 2 {sucrose [44% (w/w)], urea [22% (w/w)], 2,2′,2″-nitrilotriethanol [9% (w/w)] and Triton X-100 [0.1% (w/w)] in distilled water}; and blocking buffer {NGS [10% (v/v)] and Triton X-100 [0.5% (w/v)] in PBS}.

Tissue segments (∼100 mm^3^) were immersed in CUBIC reagent 1A (refreshed daily) for 3 days (37°C with gentle agitation). Blocking was performed overnight (4°C). All antibodies used (diluted in blocking buffer) are detailed in Table S2; staining took place (4°C) with gentle rocking. Samples were stained with primary antibodies for 4 days and secondary antibodies for 2 days. Samples were washed {PBS +Triton X-100 [0.1% (w/w)] three times (1 h at room temperature with gentle agitation) after each step. Samples were stained with DAPI (10 μM) for 2 h (room temperature) and were submerged in CUBIC reagent 2 for >24 h (37°C with gentle agitation). All tissues were imaged within 1 week; controls with no primary antibodies were used to ensure staining was antibody-specific.

### Confocal microscopy

Tissues were imaged using a Leica SP8 inverted confocal microscope with 10×/0.4 and 20×/0.75 HC PL APO objective lenses. Laser power/gain were determined manually for each fluorophore. All images are shown as maximum intensity projections using ImageJ (version 2.0.0; National Institutes of Health).

### Two-dimensional immunohistochemistry

Mammary glands were fixed in 10% neutral buffered formalin and were either stained with H&E, using standard protocols, or were subject to antigen retrieval using a PT-Link system (Agilent Technologies, Life Sciences & Chemical Analysis Group). Tissues were stained for E-cad (Table S2 used at 1:200) using a peroxidase-conjugated ImmPRESS anti-mouse IgG polymer detection kit (Vector Laboratories, MP-7402). Sections were developed using standard DAB and were counterstained with Haematoxylin.

### Statistics

For statistical analysis of myeloid cell phenotyping, the Kruskal–Wallis non-parametric test was used in conjunction with Dunn's multiple comparisons test on pre-selected column pairs (three per test). For statistical analysis between geometric MFI data, the ordinary one-way Brown–Forsythe and Welch ANOVA was used (assuming Gaussian distribution of residuals) but does not assume equal standard deviations. The Dunnett T3 post-test was performed on pre-selected column pairs (three per test), to correct for multiple comparisons.

For T cell phenotyping, the Kruskal–Wallis non-parametric test was used in conjunction with Dunn's multiple comparisons test where the mean rank of each column was compared with the mean rank in the virgin group. Additional Mann–Whitney tests were performed on an ad-hoc basis where indicated (in red); these were not corrected for multiple comparisons of data.

For statistical analysis of tumour growth rate, Mann–Whitney tests were performed at individual time points between experimental groups. *P*-values were not adjusted for multiple comparison because tests were performed independently at individual time points.

All statistical analyses followed a confidence interval of 95%, and were performed using GraphPad Prism software.

## Supplementary Material

Supplementary information

Reviewer comments
