# Supplementary material for: The immune environment of the mammary gland fluctuates during post-lactational regression and correlates with tumour growth rate
Source: Development. 2022 May 3;149(8):dev200162. doi: 10.1242/dev.200162 (PMC9124574; doi:10.1242/dev.200162)
Supplement: Supplementary information [file develop-149-200162-s1.pdf]

Fig. S1

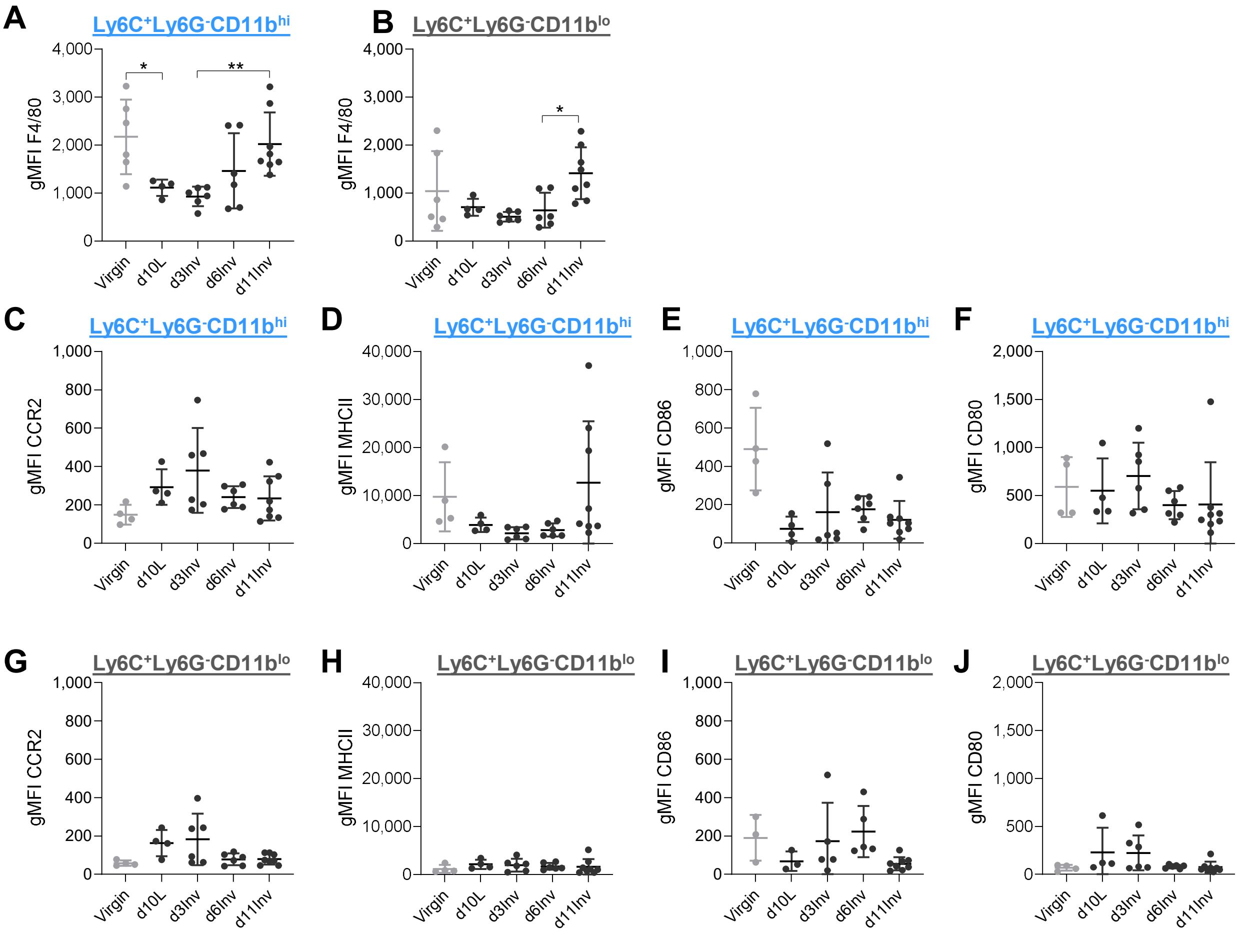

Fig. S1. Leukocytes isolated from pooled abdominal MG were analysed by flow cytometry in virgin mice and after forced involution. A-B) Geometric MFI of F4/80 expression among Ly6C$^+$Ly6G$^-$CD11b^{hi} and Ly6C$^+$Ly6G$^-$CD11b^{lo} cells. Geometric MFI of C, G) CCR2, D, H) MHCII, E, I) CD86 and F, J) CD80 expression in the indicated populations of Ly6C$^+$Ly6G$^-$ cells. All groups include 4-8 mice; (where less data points are shown, there were zero cells in the indicated population in that sample). Statistical significance (one-way Brown-Forsythe and Welch ANOVA with Dunnett T3 post-test to correct for multiple comparisons), was performed on pre-selected pairs; *P≤0.05; **P≤0.01. Dot plots show mean +/-standard deviation.

Development • Supplementary information

Fig. S2

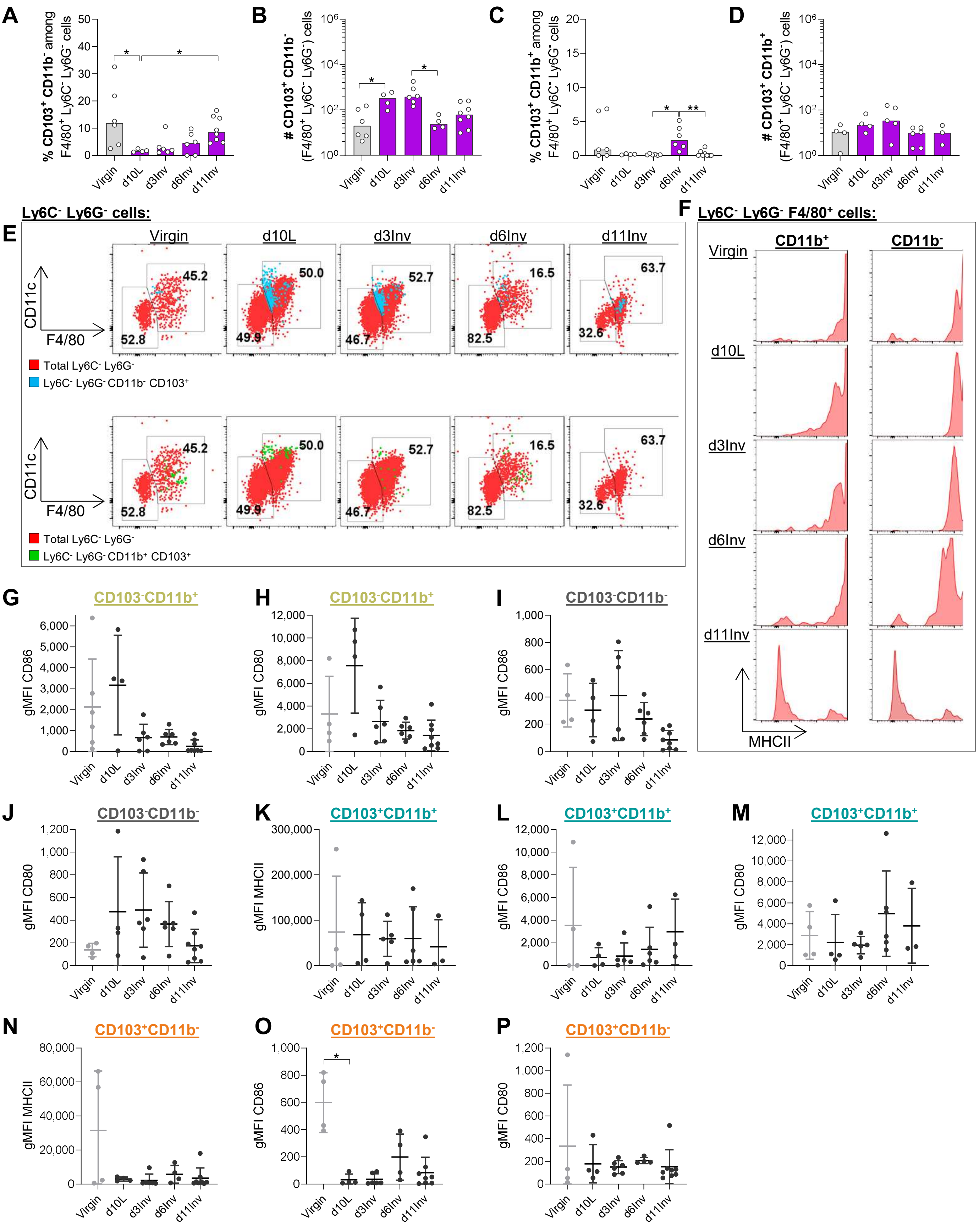

Development • Supplementary information

Fig. S2. Leukocytes isolated from pooled abdominal MG were analysed by flow cytometry after forced involution. A, C) Frequency among parent population (%) and B, D) absolute number (#) of indicated cells per pooled abdominal MG. E) Representative dot-plots demonstrating back-gating of $CD11b^{-}CD103^{+}$ cells (blue) and $CD11b^{+}CD103^{+}$ cells (green) among total $Ly6C^{-}Ly6G^{-}$ cells (red). F) Representative histograms showing MHCII expression in $Ly6C^{-}Ly6G^{-}F4/80^{+}CD11b^{+}CD103^{-}$ cells (left) and $Ly6C^{-}Ly6G^{-}F4/80^{+}CD11b^{-}CD103^{-}$ cells (right). Geometric MFI of G, I) CD86, and H, J) CD80 expression among the indicated $Ly6C^{-}Ly6G^{-}F4/80^{+}$ populations. J-P Geometric MFI of MHCII, CD86 and CD80 respectively in the indicated $Ly6C^{-}Ly6G^{-}F4/80^{+}$ populations. All groups include 4-8 mice. Statistical significance (bar charts: Kruskal-Wallis non-parametric test with Dunn's multiple comparisons test; dot plots: one-way Brown-Forsythe and Welch ANOVA with Dunnett T3 post-test to correct for multiple comparisons), was calculated on pre-selected pairs; $^{*}P\leq0.05$; $^{**}P\leq0.01$. Bar charts show medians; dot plots show mean +/-standard deviation.

Development • Supplementary information

Fig. S3

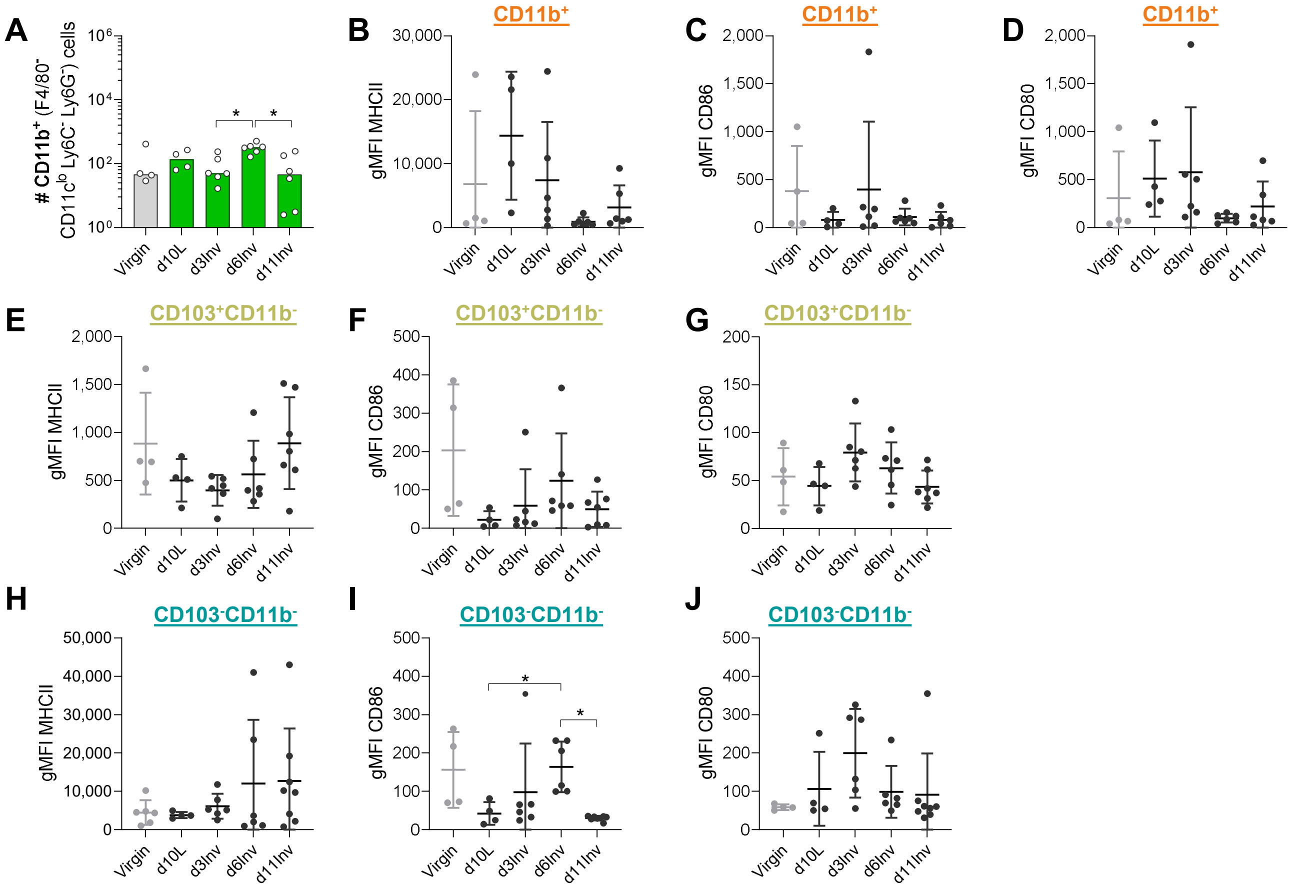

Fig. S3. Leukocytes isolated from pooled abdominal MG were analysed by flow cytometry after forced involution. A) Absolute number of indicated cells per pooled abdominal MG. B-J) Geometric MFI of MHCII, CD86 and CD80 expression among the indicated populations; B-D) $Ly6C^-Ly6G^-F4/80^-CD11c^{lo}CD11b^+$ cells; E-G) $Ly6C^-Ly6G^-F4/80^-CD11c^{lo}CD103^+CD11b^-$ cells; H-J) $Ly6C^-Ly6G^-F4/80^-CD11c^{lo}CD103^-CD11b^-$ cells. All groups include 4-8 mice. Statistical significance (bar charts: Kruskal-Wallis non-parametric test with Dunn's multiple comparisons test; dot plots: one-way Brown-Forsythe and Welch ANOVA with Dunnett T3 post-test to correct for multiple comparisons), was performed on pre-selected pairs; *P≤0.05. Bar charts show medians; dot plots show mean +/- standard deviation.

Development • Supplementary information

Fig. S4

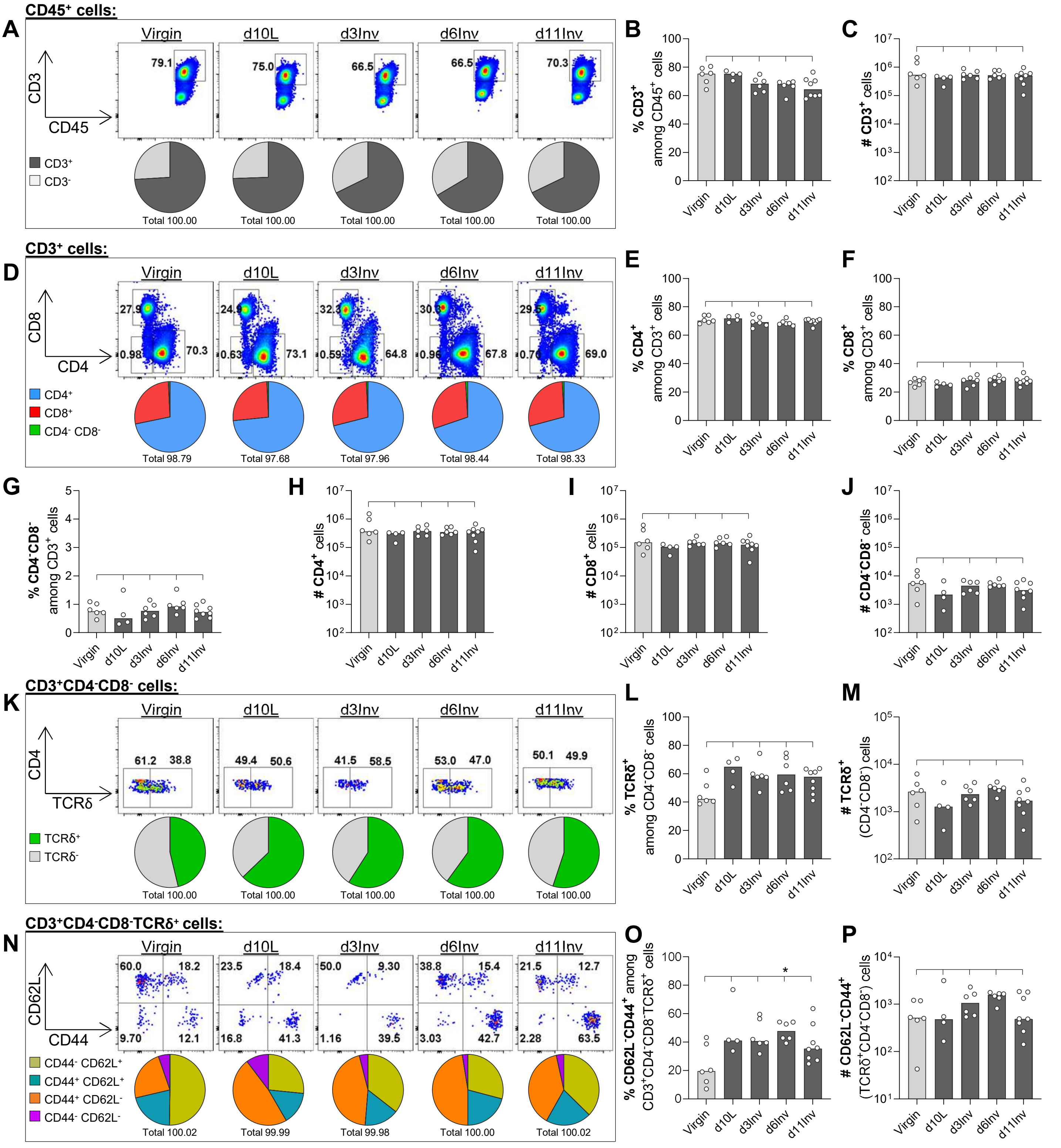

Development • Supplementary information

FFig. S4. Leukocytes isolated from dLN (inguinal) were analysed by flow cytometry after forced mammary involution. Gating strategy is shown in Fig. 4A. A, D, K, N) Representative dot-plots and pie-charts of frequencies among parent populations. B-C, E-J, L-M, O-P) Bar charts show frequencies among parent populations (%) or absolute number of cells per dLN (#) as indicated. All groups include 4-8 mice; statistical significance compared to virgin mice (black; Kruskal-Wallis test as described in methods); additional Mann-Whitney tests (red) between specific pairs indicated (*P≤0.05). Bar charts show medians.

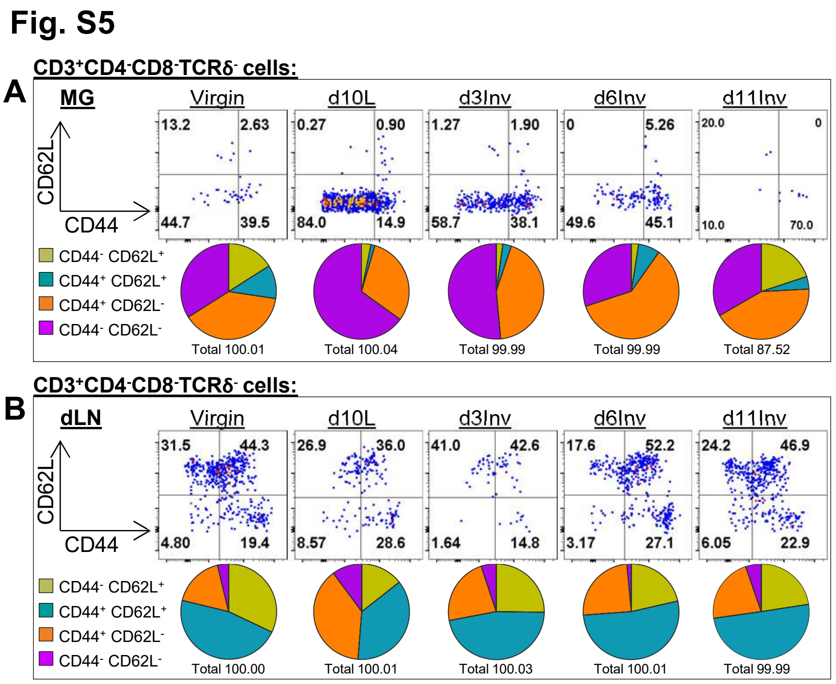

Fig. S5. Leukocytes isolated from pooled abdominal MG and dLN were analysed by flow cytometry after forced mammary involution. Representative dot-plots and pie-charts of frequencies among parent populations of the indicated populations in A) MG and B) dLN. All groups include 4-8 mice.

Development • Supplementary information

Fig. S6

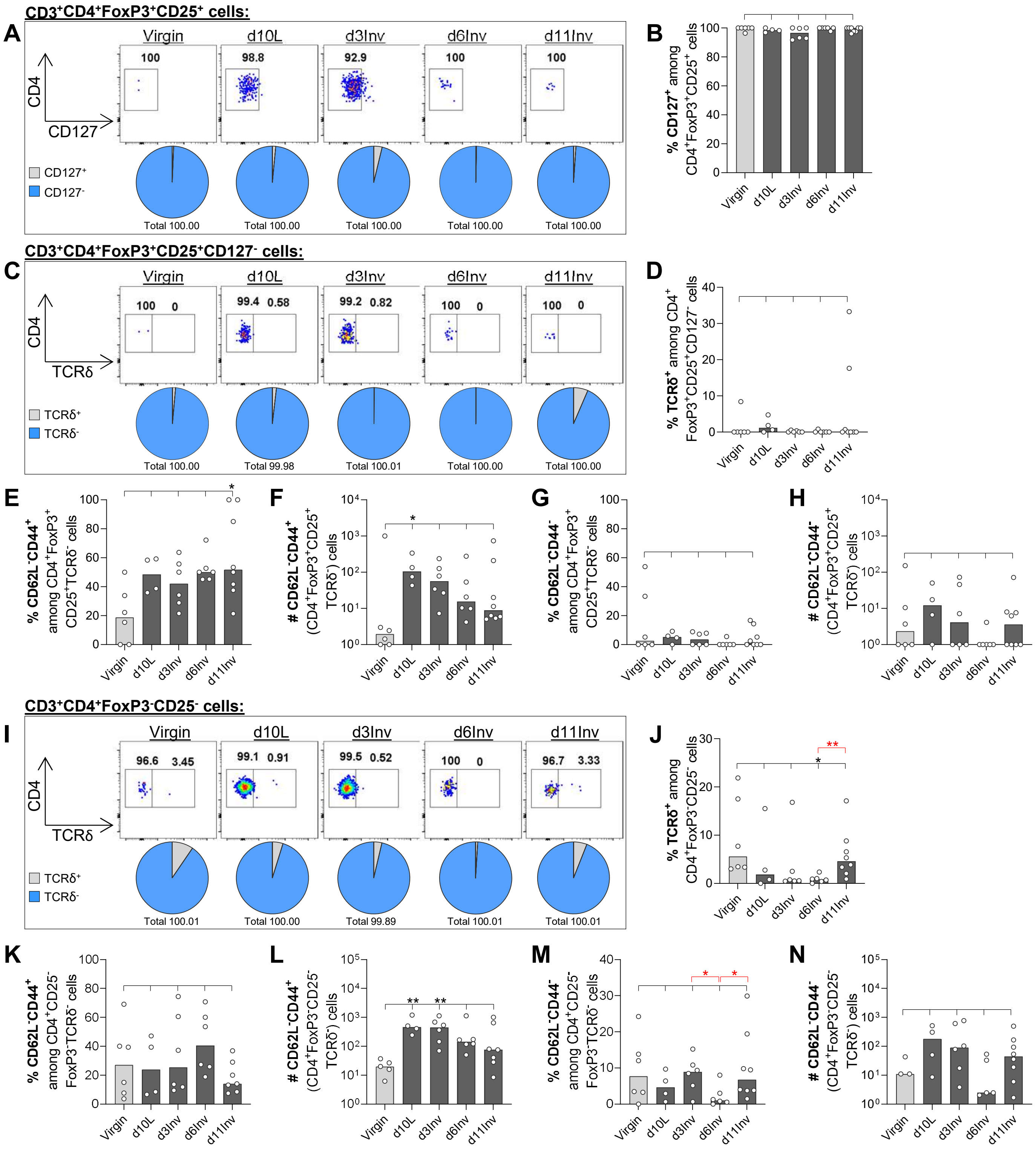

Development • Supplementary information

Fig. S6. Leukocytes isolated from pooled abdominal MG were analysed by flow cytometry after forced involution. Gating strategy is shown in Fig. 4A. A, C, I) Representative dot-plots and pie-charts of frequencies among parent populations. B, D-H, J-N) Bar charts show frequencies among parent populations (%) or absolute number of cells per pooled abdominal MG (#), as indicated. All groups include 4-8 mice; statistical significance compared to virgin mice (black; Kruskal-Wallis test as described in methods); additional Mann-Whitney tests (red) between specific pairs indicated (*P≤0.05; **P≤0.01). Bar charts show medians.

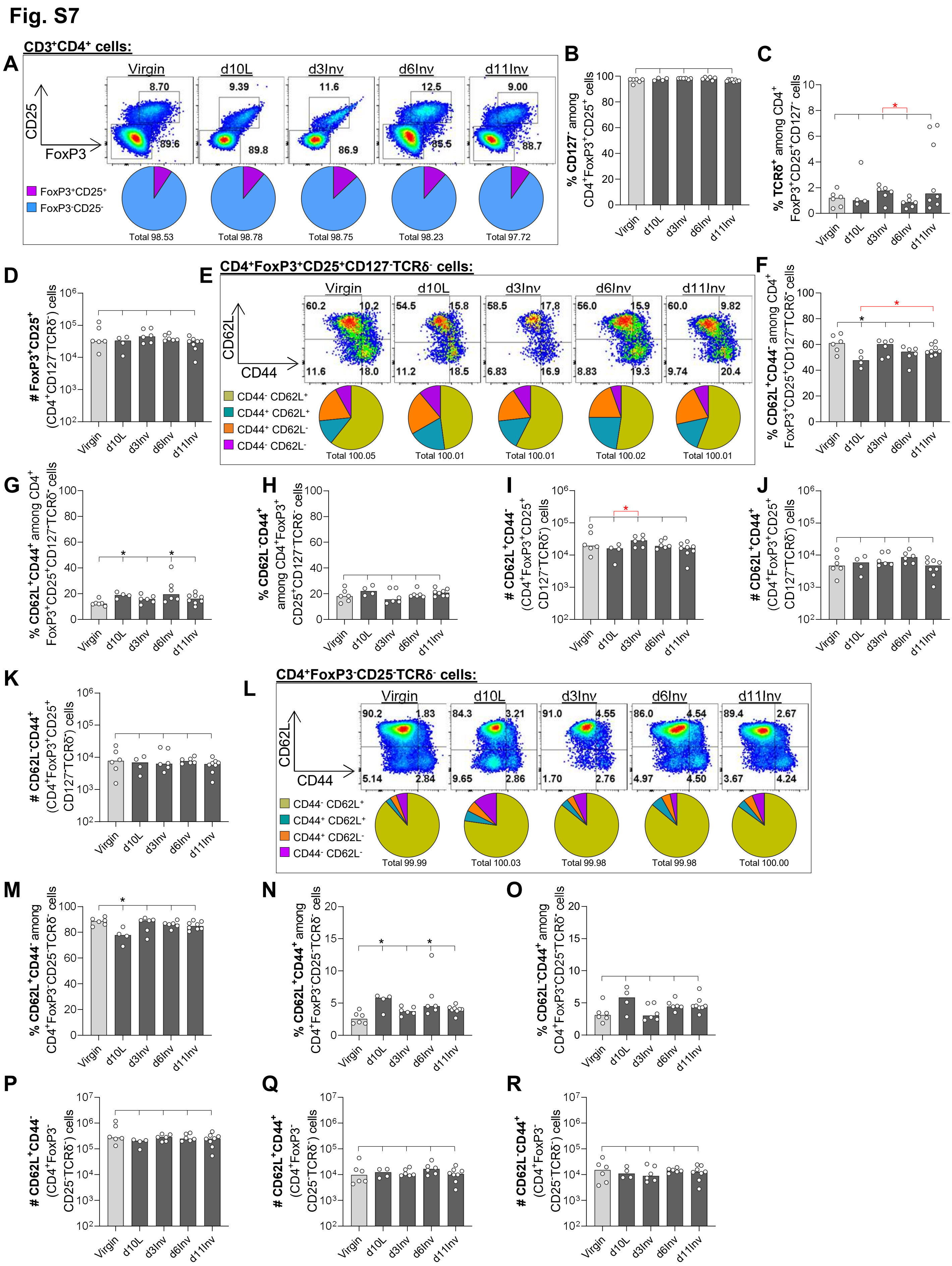

Development • Supplementary information

Fig. S7. Leukocytes isolated from dLN (inguinal) were analysed by flow cytometry after forced mammary involution. Gating strategy is shown in Fig. 4A. A, E, L) Representative dot-plots and pie-charts of frequencies among parent populations. B-D, F-K, M-R) Bar charts show frequencies among parent populations (%) or absolute number of cells per dLN (#) as indicated. All groups include 4-8 mice; statistical significance compared to virgin mice (black; Kruskal-Wallis test as described in methods); additional Mann-Whitney tests (red) between specific pairs indicated (*P≤0.05). Bar charts show medians.

Development • Supplementary information

Fig. S8

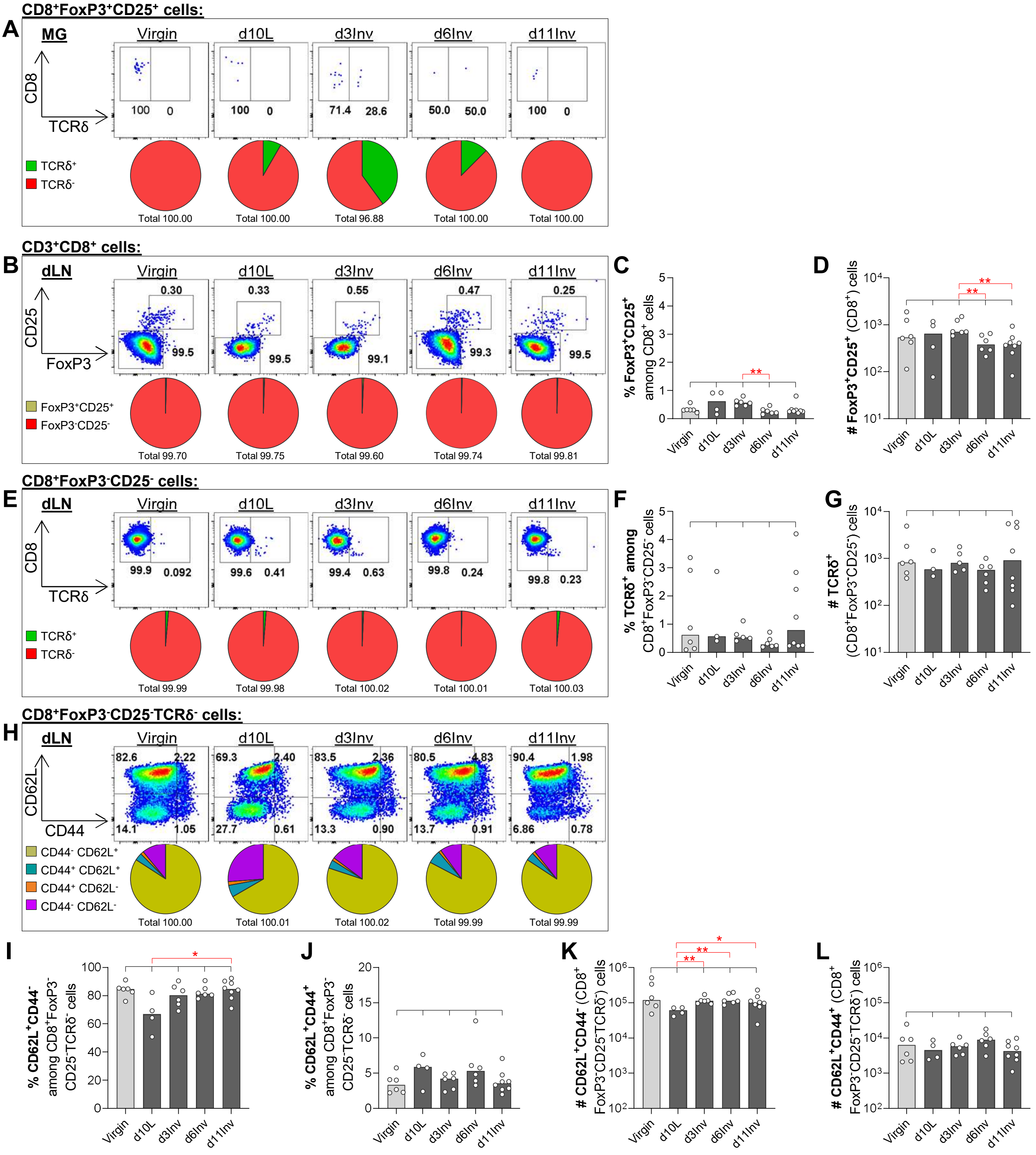

Development • Supplementary information

Fig. S8. Leukocytes isolated from pooled abdominal MG and dLN were analysed by flow cytometry after forced mammary involution. A) shows MG data, B-L) show data from the dLN. A, B, E, H) Representative dot-plots and pie-charts of frequencies among parent populations of the indicated populations. C-D, F-G, I-L) Bar charts show frequencies among parent populations (%) or absolute number of cells per dLN (#) as indicated. All groups include 4-8 mice; statistical significance compared to virgin mice (black; Kruskal-Wallis test as described in methods); additional Mann-Whitney tests (red) between specific pairs indicated (*P≤0.05; **P≤0.01). Bar charts show medians.

Development • Supplementary information

Fig. S9

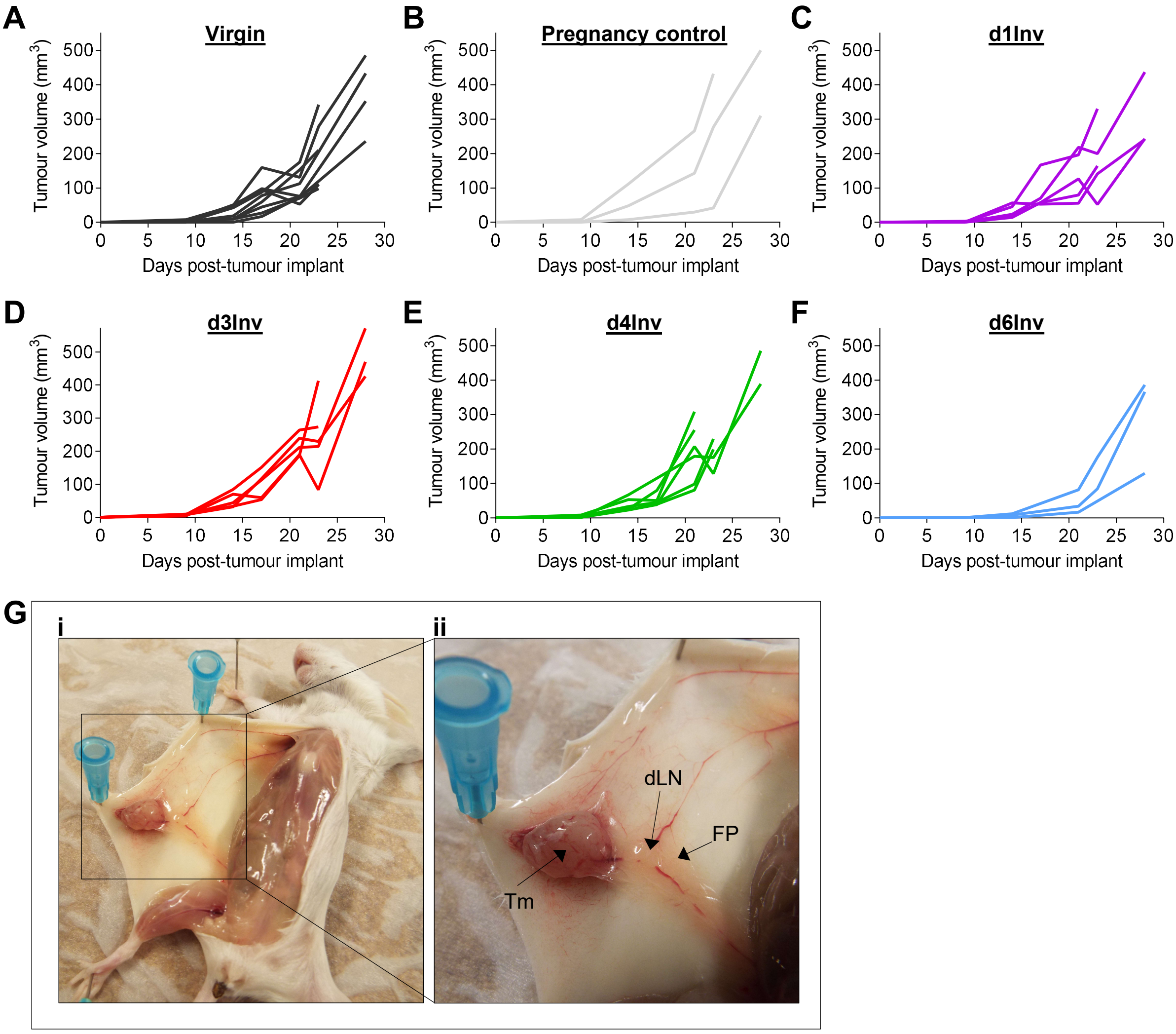

Fig. S9. Tumour growth of TUBO cells implanted at different involution time-points. Tumour volume following TUBO cell implantation into the abdominal MG of A) virgin mice, B) pregnancy control mice, or C-F) at the indicated times after forced involution; each line represents 1 mouse; groups contained 3-9 mice. G) Photograph showing the tumour implantation site in the mammary fat-pad when TUBO cells are injected non-invasively (in mice which are not pre-sedated and in which MGs are not surgically exposed). Boxed area in (i) is enlarged in (ii); Tm=tumour; FP=fat-pad; dLN= draining (inguinal) LN.

Development • Supplementary information

Fig. S10

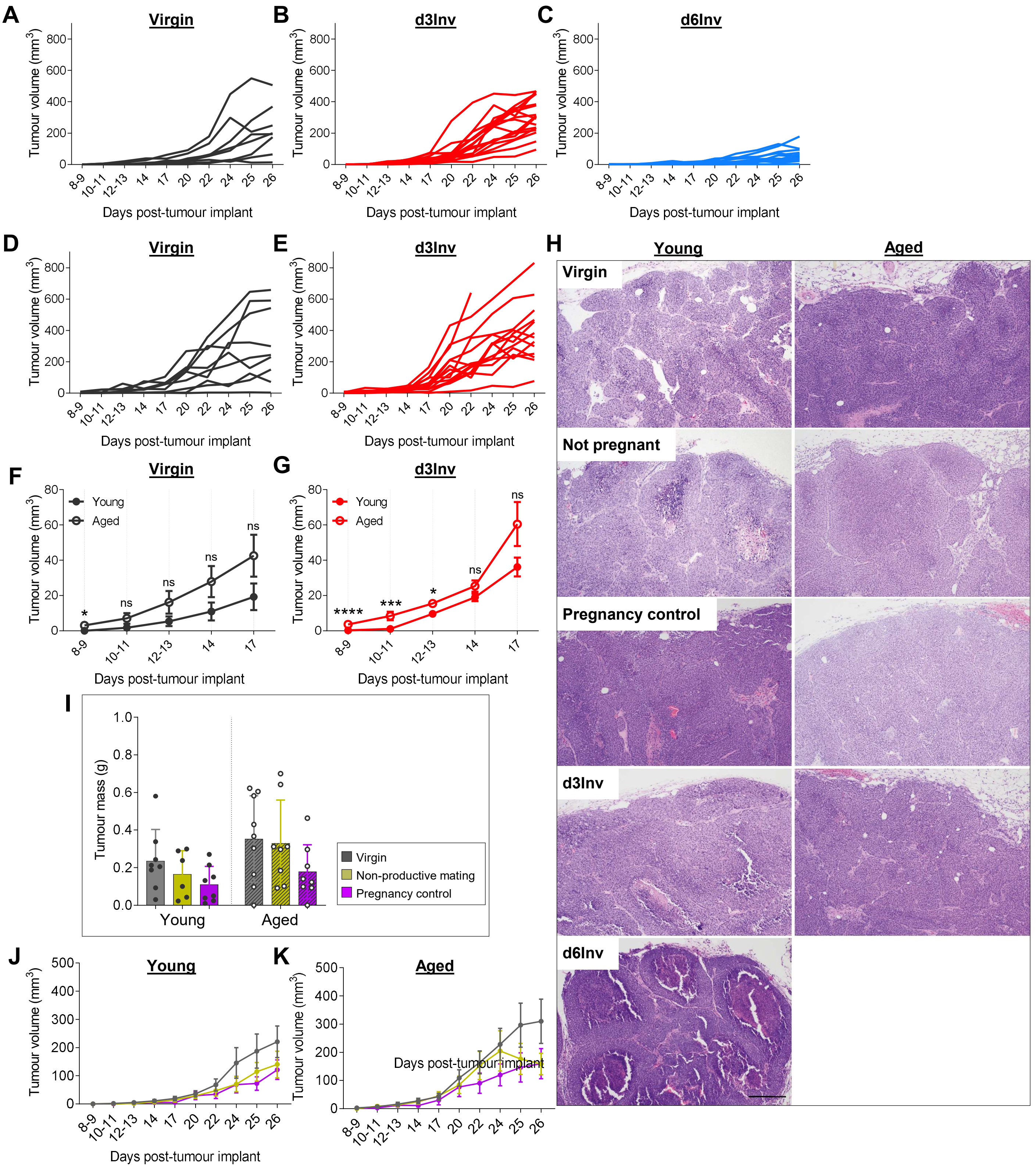

Development • Supplementary information

Fig. S10. TUBO tumour growth in young and aged mice. Young (6-8 week) (A-C) and aged (10 month) (D-E) BALB/c mice were mated and TUBO cells were implanted into the abdominal MG at the indicated time-points post-forced involution, or into MG of virgin mice. Tumour volume (individual mice) in A, D) virgin; B, E) d3Inv; and C) d6Inv mice. F-G) Mean tumour volumes in virgin and d3Inv mice respectively; *these data are shown in Fig. 8H-I, but have been replotted here to enable visualisation of the initial tumour growth*. H) 2D H&E images of tumours harvested 26 days post-implantation; scale bar =200µm. I) Mean tumour mass at 26 days post-implant and J-K) mean tumour volumes in young and aged mice respectively of control groups. Bar charts show medians; tumour growth (by volume) where each line = 1 mouse (A-E) and presented as mean+/-SEM (F-G, and J-K). Groups contained 8-17 mice; statistical significance: Mann-Whitney tests performed at individual time-points; (*P≤0.05; ***P≤0.001, ****P≤0.0001; ns= non-significant).

Table S1. List of antibodies used for flow cytometry

Target	Clone	Fluorophore	Dilution	Source	Catalog number
CD16/CD32	93	Purified	1:200	BioLegend	101302
CD45	30-F11	BV785	1:200	BioLegend	103149
CD3ε	145-2C11	FITC	1:200	BioLegend	100306
CD4	RM4-5	BV421	1:300	BioLegend	100543
CD8α	53-6.7	APC	1:300	BioLegend	100712
Foxp3	FJK-16S	PE	1:200	Invitrogen	12-5773-82
CD25	PC61	PE Dazzle	1:200	BioLegend	102048
CD127	A7R34	APC Cy7	1:200	BioLegend	135040
CD44	IM7	PE Cy5	1:500	BioLegend	103009
CD62L	MEL-14	BV605	1:200	BioLegend	104438
TCR-γ/δ	GL3	BV650	1:200	BD Horizon	563993
Gr1	RB6-8C5	BUV395	1:200	BD Horizon	563849
CD11c	HL3	BUV737	1:300	BD Horizon	564986
F4/80	BM8	BV421	1:100	BioLegend	123132
Ly6C	HK1.4	BV605	1:300	BioLegend	128036
CD11b	M1/70	BV650	1:400	BioLegend	101259
CD103	2E7	FITC	1:200	BioLegend	121420
Ly6G	1A8	PE Dazzle	1:400	BioLegend	127648
CD80	16-10A1	PE Cy5	1:300	BioLegend	104712
MHCII	M5/114.15.2	APC	1:300	Invitrogen	17-5321-81
CD86	GL-1	APC Cy7	1:400	BioLegend	105029

Table S2. List of antibodies used for imaging

Target	Host species	Target species	Fluorophore	Dilution	Clone	Source	Catalog number
Smooth muscle actin-α	Rabbit	Mouse	Unconjugated	1:300	Polyclonal	Abcam	ab5694
E-cadherin	Rabbit	Mouse	Unconjugated	1:50	24E10	Cell Signaling Technology	3195
CD45	Rat	Mouse	Unconjugated	1:300	30-F11	BioLegend	103102
IgG (H+L)	Goat	Rabbit	AlexaFluor-488	1:500	Polyclonal	Invitrogen	A11008
IgG (H+L)	Goat	Rat	AlexaFluor-647	1:500	Polyclonal	Invitrogen	A21247
IgG (H+L)	Goat	Rat	Cy3	1:500	Polyclonal	Invitrogen	A10522

Development • Supplementary information
