# Supplementary material for: The immune environment of the mammary gland fluctuates during post-lactational regression and correlates with tumour growth rate
Source: Development. 2022 May 3;149(8):dev200162. doi: 10.1242/dev.200162 (PMC9124574; doi:10.1242/dev.200162)
Supplement: Reviewer comments [file develop-149-200162_review_history.pdf]

The immune environment of the mammary gland fluctuates during post-lactational regression and correlates with tumour growth rate

Jessica Hitchcock, Katherine Hughes, Sara Pensa, Bethan Lloyd-Lewis and Christine J. Watson

DOI: 10.1242/dev.200162

Editor: Florent Ginhoux

Review timeline

Original submission:	3 September 2021
Editorial decision:	1 October 2021
First revision received:	26 January 2022
Editorial decision:	14 February 2022
Second revision received:	17 March 2022
Editorial decision:	30 March 2022
Third revision received:	1 April 2022
Accepted:	4 April 2022

Original submission

First decision letter

MS ID#: DEVELOP/2021/200162

MS TITLE: The immune environment of the mammary gland fluctuates during post-lactational regression and is associated with altered tumorigenicity

AUTHORS: Christine J Watson, Jessica Hitchcock, Katherine Hughes, Sara Pensa, and Bethan Lloyd-Lewis

I have now received all the referees' reports on the above manuscript, and have reached a decision. The referees' comments are appended below, or you can access them online: please go to BenchPress and click on the 'Manuscripts with Decisions' queue in the Author Area.

As you will see, the referees express considerable interest in your work, but have some significant criticisms and recommend a substantial revision of your manuscript before we can consider publication. If you are able to revise the manuscript along the lines suggested, which may involve further experiments, I will be happy receive a revised version of the manuscript. Your revised paper will be re-reviewed by one or more of the original referees, and acceptance of your manuscript will depend on your addressing satisfactorily the reviewers' major concerns. Please also note that Development will normally permit only one round of major revision.

We are aware that you may be experiencing disruption to the normal running of your lab that make experimental revisions challenging. If it would be helpful, we encourage you to contact us to discuss your revision in greater detail. Please send us a point-by-point response indicating where you are able to address concerns raised (either experimentally or by changes to the text) and where you will not be able to do so within the normal timeframe of a revision. We will then provide further guidance. Please also note that we are happy to extend revision timeframes as necessary.

Please attend to all of the reviewers' comments and ensure that you clearly highlight all changes made in the revised manuscript. Please avoid using 'Tracked changes' in Word files as these are lost

© 2022. Published by The Company of Biologists under the terms of the Creative Commons Attribution License (https://creativecommons.org/licenses/by/4.0/).

in PDF conversion. I should be grateful if you would also provide a point-by-point response detailing how you have dealt with the points raised by the reviewers in the 'Response to Reviewers' box. If you do not agree with any of their criticisms or suggestions please explain clearly why this is so.

Reviewer 1

Advance summary and potential significance to field

see below

Comments for the author

In the current manuscript, Hitchcock et al. analyzed the immune compartment in the mammary gland in virgin mice, lactating mice and at 3 time points during involution. They describe that the immune cells in the mammary gland change in numbers at the different time points. They further implanted tumor cells at different time points and found that at day 3 of involution tumors grew faster whereas at day 6 of involution they grew slower, which was also observed in aged mice. While it is potentially an interesting manuscript, the conclusions are not supported by their data. The immune cells at the different time points have not been characterized well and have not been analyzed in the tumors at all.
No conclusion can be drawn on the temporal changes of immune cells as many populations could not be adequately identified (see below). In particular the myeloid cell characterization is not of sufficient quality.
The authors also mention changes in gd T cells but do not elaborate further.
Overall, an extensive immune profiling is required at the different involution time points to decipher which cells could be key players in the process of involution and tumor growth. Also in the tumor microenvironment immune cell phenotyping is needed at the different time points after tumor implantation. In addition, immune cell localization should also be assessed in more detail (not only CD45+ cells).
It is not clear why they did the 'forced weaning' on d10 post-partum and not at a time point when the pups would actually be weaned. Different time points during lactation have a different immune cell composition which could influence the involution and tumor growth. This should be clarified. Ideally, they should repeat the experiments with a later time point of 'forced weaning'.

Specific comments:
Fig. 1. On d6Inv it seems that CD45+ cells are not associated to alveoli anymore. Can they comment on this?
Fig. 2, the gating strategy is not clear. Why do they define gd T cells as cells pre-gated on CD44+ cells? Also the TCRb staining does not appear to have worked. Can they show an FMO? According to their gating strategy, TCRgd+ cells would express TCRb.
Fig. 3, there are several issues also in this gating strategy and flow cytometry analysis:
A gate is drawn through the middle of a CD11b- population defining CD11blo and CD11b- cells. These cells are all clearly CD11b- cells. Gr1+ (Ly6C+) CD11b- cells are not myeloid cells and likely T cells.
Two resident macrophages have been described in virgin mice (Jäppinen et al. 2019) based on F4/80 and CD11b expression. Here, F4/80 does not seem to have worked. They state for example that in virgin mice 80% of CD11bhi cells are F4/80+ (Fig. 3Q). Representative FACS plots should be shown for this as the F4/80 staining in Fig. 3A is not convincing. Thus, using their gating strategy dendritic cells and macrophages cannot be distinguished. Of note, a recent report (Dawson et al. 2020) describes ductal macrophages as CD11bloCD11c+ cells. These macrophages are likely the MHCIIhi population shown in Fig. 4D, which the authors claim to be dendritic cells. But also in this Figure, CD11c is not a convincing staining and an FMO should be included.
MDSCs is an outdated term and should not be used here. These cells are monocytes and neutrophils.
Tumor experiments:
Figure 5. Differences in tumor growth appear to be very small between the different time points. Is it significant? Fig. 5H – Fig. 5J is the same experiment just different groups shown. This should be clarified. It is unclear whether the experiment was performed once or whether it has been

© 2022. Published by The Company of Biologists under the terms of the Creative Commons Attribution License (https://creativecommons.org/licenses/by/4.0/).

repeated. The number of mice used for some groups is very small. For example, d6Inv is only n=3 (Suppl. Fig 2). This is not enough to make a statement that tumor growth is slower in this group. They claim that tumors grew faster when TUBO cells were implanted at d3Inv and slower when implanted at d6Inv while they also claimed that d4Inv is similar to d1Inv. What is the difference in immune composition between d3Inv and d4Inv where a difference in tumor growth was observed? What are the differences in immune cells in the different groups at different time points of tumor growth?
Fig. 6, the tumor cell administration is not entirely clear compared to Fig. 5. They call it subcutaneous implantation. This is confusing as tumor cells were injected into the mammary gland and not s.c.
Fig. 6G-H, again, these are the graphs from C and D shown in one graph. This should be explained. Have these experiments (young vs. old) been performed together or are these pooled data of several independent experiments?

Reviewer 2

Advance summary and potential significance to field

This paper quantifies lymphoid and myeloid populations throughout mouse mammary gland involution and compares the growth of mammary tumours implanted at different stages of involution and though ageing. The changes in cancer risk associated with reproduction are important to better understand and the impact of the immune environment on this is an important area of study. Using FACS analysis during involution, the authors revealed changes in Tregs and CD4-CD8- T cells, neutrophil/monocytes populations and some CD11c+ populations of unclear identity. Tumour growth was analysed after implantation at different stages of involution and ageing. Faster growth was observed with implantation at 3 days involution compared to virgin mice and slower growth with implantation at day 6 although the reason for this was not explored. Overall, the study presents some interesting and novel findings, but these are mostly preliminary and are not well-connected. The impression is given in the title, abstract and introduction that changes in immune cell frequency may impact tumour growth, but this is not addressed experimentally. The cohesiveness of the study and the strength of the involution/immune/tumour connections would be improved by a more refined analysis of myeloid populations, assessing immune population interaction with tumours by imaging, and assessing the impact of the altered immune cells on tumour. These would require very significant additional experimental work and modifications to the current paper.

Comments for the author

Major points
The myeloid cell populations analysed are not well defined. Gr1 stains both Ly6G and Ly6C, so the emphasis on this marker is not helpful. Use of Ly6C, Ly6G CD11b and MHCII to define previously characterised monocyte and neutrophil populations would be better and more standard.
The analysis macrophage and dendritic cell populations is unclear and does not make use of current methods for their identification. Eg DCs: CD11c+MHCII+ (excluding macrophages) and separation of these into cDC1/2 using a selection of CD103/CD11b/Xcr1/CD172a. Macrophages should be identified with an additional marker such as CD64 or MerTK. F4/80 alone is not sufficient. These methods are well-established and widely accepted (see Guilliams et al, 2016, Immunity).
The imaging does not add significantly to the paper and not quantified or connected to the immune populations analysed. Immune cell distribution should be quantified to back up statements made in the text. Imaging of CD45 and SMA (F1A) during involution is already published by the authors in the FEBS journal 2020 so does not provide new information in this paper.
The immune characterisation and tumour analysis are not connected in this study as is implied by the title and abstract. Functional data for some immune subsets are needed eg. Does the change between day 3 and 6 exist in mice depleted of neutrophils by anti-Ly6G, or macrophages by anti-Csf1r? Which immune populations interact with the tumour upon implantation to implicate them in rejection?

© 2022. Published by The Company of Biologists under the terms of the Creative Commons Attribution License (https://creativecommons.org/licenses/by/4.0/).

Minor points
The lymphoid cell analysis is thorough and the observed changes in T cell subsets are interesting, but these could be analysed and presented in a more intuitive way for readers to understand. Eg. Pie charts that show global shifts over the different stages. The analysis would be clearer if individual populations were separated, then activation markers examined on these. Aligning the different organ analyses and moving the relative % measurements to the supps would aid interpretation.
F3: dots are too small for colours to be seen
F3A Gr1- CD11b–/low looks like a single population
F3Q-S: low macrophage percentage does not fit with the known abundance of macrophages in the mammary gland, especially in lactation
F6A-B: were use of Bl/6 vs Balb/c studs controlled for in this data?
F6B: define TLL
F6E-F: label young/old
F6D/F, C/E: data should only be shown once
F6F,I: clearly define ND, this is often used as not detected
Line 254: define monocytic myeloid cells
Lines 138: some of these references are not appropriate for preclinical cancer models during involution

Methods:
Specify the media that the tumour cells injected in.
Line 698: reference does not use Ficoll gradient cell enrichment Specify density centrifugation speeds Does Ficoll enrichment change population frequencies?
Line 735: Define NBF

First revision

Author response to reviewers' comments

Dear Florent

Thank you for providing us with the reviewers' comments and for giving us an opportunity to submit a revised version of our manuscript. We provide a point-by- point response to the reviewers' comments below. However, I would like to preface this with a statement on our circumstances. I retired from my academic post at the University of Cambridge (on the 30th September 2021) and my lab is now completely closed down. I no longer have grant funding or the resources to carry out substantial experiments and since we used aged mice for some of the work, the time scale would extend beyond 12 months. Furthermore, my laboratory was closed from March 2020 due to COVID restrictions and all the authors on this manuscript are now employed elsewhere. So, as I'm sure you will appreciate, we are not in a position to carry out much in the way of additional experiments. I hope you understand the difficulties this presents to us.

We are extremely grateful for the detailed comments provided by the reviewers', and our response to these is as follows:

Reviewer 1 Advance Summary and Potential Significance to Field:

While it is potentially an interesting manuscript, the conclusions are not supported by their data. The immune cells at the different time points have not been characterized well and have not been analyzed in the tumors at all.

We have fully taken this feedback onboard and have re-analysed the entire immune cell characterisation section of the study. We have provided representative dot plots for all time-points examined in both the mammary gland (MG) and draining LN (dLN), whereas previously we had only provided snapshots at day 3 post-involution. This therefore provides a much more detailed picture of our data.

© 2022. Published by The Company of Biologists under the terms of the Creative Commons Attribution License (https://creativecommons.org/licenses/by/4.0/).

The reviewers' very kindly offered suggestions as to how we may gate our data differently and have highlighted several key publications which we did not cite originally. These insights have greatly benefitted our revised analysis and have ensured the robust interpretation of our observations. Moreover, we have presented much of the data both as pie-charts and as histograms. This enables readers to quickly visualise the main differences, with the histograms detailing inter-mouse variability in a clear and transparent manner. We have coupled this with absolute cell numbers and so we feel that we have provided a fully comprehensive analysis of the available data.

We acknowledge that we have not characterised the intratumoral immune compartment at all by flow cytometry. However, in this study we sought to identify associations between the mammary immune environment and the potential effect this had on the initial outgrowth of implanted tumour cells. We have reworded several passages of text to highlight this. Whilst we agree it would be interesting to address the changes in immune cells within the tumours implanted at different stages of involution, we would argue that tumours modulate their microenvironment as they progress and so analysis of immune cells present in the tumours at the end point is not relevant to the specific question we are asking i.e. how do the different immune cell populations present in the mammary gland at specific times of involution affect initial tumour growth?

Lastly, we agree that we have not proven the connection between the immune cells present at the different time points of involution and the different rates of tumour growth at these time points. While our data are correlative and suggest that specific immune cell types may have an impact on tumour cell growth, we agree that they are not conclusive. We have significantly re-written the manuscript to make this correlative connection more clear and have toned-down our conclusions accordingly.

No conclusion can be drawn on the temporal changes of immune cells as many populations could not be adequately identified (see below). In particular the myeloid cell characterization is not of sufficient quality.

As outlined above, we have taken onboard the reviewers' expert comments and have regated the immune cell characterisation to better identify populations. For example, we changed our myeloid gating strategy so that it is more in line with published data (Japinnen et al. 2019), whereby we select populations on expression of F4/80 prior to characterisation using other markers. We do appreciate that using a 12-colour flow panel we are not able to "adequately identify" myeloid cells to the sufficient level of detail that the reviewers have suggested. However, this was not the scope of our study; we sought to identify whether changes in immune cells married up with changes in initial tumour growth rate. We believe our data suggest there is a correlation and it will be fascinating to identify the intricacies of this relationship in future studies.

We did make particular effort to categorise immune cells according to their surface marker expression rather than formally naming different cell types (e.g. a DC or a neutrophil); we agree that with surface marker identification alone we are unable to make these distinctions. However, what is clear is that there are temporal changes in immune cells types and numbers relative to each other, and these are adequately analysed for the purpose of this study. We have tried to rewrite the text to reflect this.

The authors also mention changes in $\gamma\delta$ T cells but do not elaborate further.

We agree that further analysis of $\gamma\delta$ T cells would be interesting but is beyond the scope of this work. As above, we only sought to make associations between the immune profile at different stages of involution and whether this corresponds to tumour growth. We did not expect such distinct changes in $\gamma\delta$ T cells and agree that a more thorough characterisation is required (especially as we did not have adequate markers in our flow panel to sufficiently identify the plethora of unconventional T cells present). It would be interesting to dissect the functional behaviours of these cells by detailed characterisation and functional studies in future work.

Overall, an extensive immune profiling is required at the different involution time points to decipher which cells could be key players in the process of involution and tumor growth. Also in the tumor microenvironment, immune cell phenotyping is needed at the different time points

© 2022. Published by The Company of Biologists under the terms of the Creative Commons Attribution License (https://creativecommons.org/licenses/by/4.0/).

after tumor implantation.
We respectfully suggest that our revised analysis is adequate to identify at least some of the key players. As discussed above, the immune cells present in the tumour at different times during tumour growth (although of interest) are not relevant to our question. Furthermore, a substantial increase in the numbers of mice used would be required. Apart from the time and expense, it is not ethically justified to subject more mice to considerable harm than necessary.

In addition, immune cell localization should also be assessed in more detail (not only CD45+ cells).

We agree that localisation of some of the identified cells could be intriguing. However, because so many of the populations in question share surface marker expression, we think that we would not be able to sufficiently identify cells histologically, thus in the context of this paper, it may add little value.

It is not clear why they did the 'forced weaning' on d10 post-partum and not at a time point when the pups would actually be weaned.

This lactation time point is standard in the field and is representative of the peak in lactation, when alveoli are fully developed, and, when litter sizes are normalised to 6- 8 pups (as they were in this study), all MGs are being equally suckled. This therefore enables a synchronous involution across all glands within the mouse, and enables comparison between mice. We have been carrying out forced weaning experiments on mice for over two decades and this is a widely accepted procedure. Gradual ‘natural’ weaning makes comparison between mice difficult as different regions of the gland remodel at different rates. In addition, many women undergo ‘forced weaning’ for various reasons and so this model is entirely relevant.

Different time points during lactation have a different immune cell composition, which could influence the involution and tumor growth. This should be clarified.

Ideally, they should repeat the experiments with a later time point of 'forced weaning'.

This is true and this is why we chose a specific lactation time point, that of maximum milk production, ensuring no regions of the gland are undergoing involution before the forced weaning process. We are not sure if the reviewer means a later time point of lactation at which to initiate the forced weaning. We disagree that it would be worthwhile to do this as once the peak of lactation has passed, the gland undergoes a gradual, multifocal involution and not all glands will involute in a similar way.

Specific comments:
Fig. 1. On d6Inv it seems that CD45+ cells are not associated to alveoli anymore. Can they comment on this?

By d6Inv, very few alveoli remain following the extensive cell death of the secretory alveolar epithelium, while the myoepithelial cells contract and realign towards the ducts (as shown in the imaging in Figure 1). We have clarified this in the text.

Fig. 2, the gating strategy is not clear. Why do they define γδ T cells as cells pre- gated on CD44+ cells?

We did originally gate on potentially activated (CD44+) γδ cells because we thought these cells may be contributing to the involution process i.e. we were trying to look at γδ cells which may have a function rather than simply being bystander cells. We did not classify γδ cells as CD44+. However, we acknowledge that this gating does not consider all γδ T cells (i.e. CD44- cells) and thus we have revised the gating strategy used.

Also, the TCRb staining does not appear to have worked. Can they show an FMO? According to their gating strategy, TCRgd+ cells would express TCRb.

We agree the TCRb staining does not add to our interpretation and we have excluded this data from our revised analysis.

© 2022. Published by The Company of Biologists under the terms of the Creative Commons Attribution License (https://creativecommons.org/licenses/by/4.0/).

Fig. 3, there are several issues also in this gating strategy and flow cytometry analysis:A gate is drawn through the middle of a CD11b- population defining CD11blo and CD11b- cells.

In our original submission, we only presented dot plots from one timepoint and we fully acknowledge that this sometimes makes gating difficult to justify. We are grateful to reviewer 1 for highlighting this and have now presented representative dot plots for all timepoints which circumvents this issue.

These cells are all clearly CD11b- cells. Gr1+ (Ly6C+) CD11b- cells are not myeloid cells and likely T cells.

We completely agree that Ly6C+CD11b- cells are likely to be T cells and we have tried to reference this in our revised gating strategy of these cells (see revised text).

As above, the provision of dot plots throughout the timecourse should help in justification of the gating used.

Two resident macrophages have been described in virgin mice (Jäppinen et al. 2019) based on F4/80 and CD11b expression. Here, F4/80 does not seem to have worked.

We thank reviewer 1 for highlighting the Jäppinen *et al*. study on intramammary macrophage populations as it is extremely insightful. We have now changed our gating strategy, choosing to distinguish between F4/80+/- cells first, prior to gating for other markers including CD11b (originally we gated first on CD11b/Gr1). This has enabled much more robust interpretation of F4/80 expression and we hope the reviewers agree from the representative dot plots that the F4/80 staining has worked.

They state for example that in virgin mice, 80% of CD11bhi cells are F4/80+ (Fig. 3Q). Representative FACS plots should be shown for this as the F4/80 staining in Fig. 3A is not convincing.

The FACS gating has been revised and all necessary timepoints are now shown.

Thus, using their gating strategy dendritic cells and macrophages cannot be distinguished. Of note, a recent report (Dawson et al. 2020) describes ductal macrophages as CD11bloCD11c+ cells. These macrophages are likely the MHCIIhi population shown in Fig. 4D, which the authors claim to be dendritic cells.

Macrophages in the MG are known to co-express F4/80 and CD11c and also MHCII. Due to the limitations of our 12 colour flow panel, we have endeavoured not to distinguish between macrophages and DCs, rather describing cells by their surface marker expression. We have clarified this in the revised text. We agree that MHCIIhi CD11c+ cells may include macrophages and DCs.

But also in this Figure, CD11c is not a convincing staining and an FMO should be included.

As above, we have reanalysed this data and the provision of additional timepoints enables more reliable interpretation of the staining (see Fig 2U and 3L).

MDSCs is an outdated term and should not be used here. These cells are monocytes and neutrophils.

We respectfully disagree that MDSC is an outdated term; and we were trying to emphasise that the Ly6C+ cells could be monocytes of pro-inflammatory or suppressive nature. However, we have changed the terminology and make no reference to MDSC.

Tumor experiments:
Figure 5. Differences in tumor growth appear to be very small between the different time points. Is it significant? Fig. 5H – Fig. 5J is the same experiment just different groups shown. This should be clarified. It is unclear whether the experiment was performed once or whether it has been repeated. The number of mice

© 2022. Published by The Company of Biologists under the terms of the Creative Commons Attribution License (https://creativecommons.org/licenses/by/4.0/).

used for some groups is very small. For example, d6Inv is only n=3 (Suppl. Fig 2). This is not enough to make a statement that tumor growth is slower in this group.

We thank the reviewer for highlighting the difficulties in interpreting this data. We have now included separate statistical analysis at each time point during tumour growth so that any differences are clearly identifiable. We have re-written the text to reflect where data is from the same experiment.

We agree that the numbers of mice are small in some groups and acknowledge this is unfortunate. These are logistically extremely difficult experiments to perform as mice do not always become pregnant, do not litter when expected, and do not always have consistent litter sizes. Therefore, a large number of spare pups must be ready at precisely the right time so that these can be cross-fostered within 2 days of birth. In addition, this project was funded by a small project grant and there was insufficient funding for much larger studies. We therefore strived to ensure that all groups comprised of at least 3 mice but we agree that not all groups are of equal size and that this is a practical restraint of performing these types of experiments.
Given the strong trends in the data, we would expect statistical significance to be reached at more time points.

They claim that tumors grew faster when TUBO cells were implanted at d3Inv and slower when implanted at d6Inv while they also claimed that d4Inv is similar to d1Inv. What is the difference in immune composition between d3Inv and d4Inv where a difference in tumor growth was observed?

We have not shown these data. However, the largest change in tumour growth was between d3Inv and d6Inv and so we have rewritten the text to emphasise this.

What are the differences in immune cells in the different groups at different time points of tumor growth?

Please refer to our discussion above. .

Fig. 6, the tumor cell administration is not entirely clear compared to Fig. 5. They call it subcutaneous implantation. This is confusing as tumor cells were injected into the mammary gland and not s.c.

We apologise for this confusion. This terminology was used to emphasise that it was not a surgical procedure; the injections into the MG performed without sedation were performed similarly to a s.c. injection. We have changed the text to remove this confusion.

Fig. 6G-H, again, these are the graphs from C and D shown in one graph. This should be explained. Have these experiments (young vs. old) been performed together or are these pooled data of several independent experiments?

Again, we apologise for the confusion. This was one large experiment where young and aged mice were compared in parallel at the different time-points described. We have changed the text to clarify these points. .

Reviewer 2 Advance Summary and Potential Significance to Field:
Overall, the study presents some interesting and novel findings, but these are mostly preliminary and are not well-connected. The impression is given in the title, abstract and introduction that changes in immune cell frequency may impact tumour growth, but this is not addressed experimentally.

We agree with these comments and fully acknowledge that this study does not provide direct evidence that tumour growth is altered by particular immune subsets. The amount of work required to draw such mechanistic conclusions, including depleting different types of immune cells as is substantial and currently not feasible with our resources.

However, what we are suggesting is that the immune cells present at the time of tumour cell implantation affect the initial growth of tumours. Our data provide strong correlative evidence of

© 2022. Published by The Company of Biologists under the terms of the Creative Commons Attribution License (https://creativecommons.org/licenses/by/4.0/).

this and we suggest that these are nevertheless important observations. We have changed the title and text to reflect that these are correlative data and not conclusive.

The cohesiveness of the study and the strength of the involution/immune/tumour connections would be improved by a more refined analysis of myeloid populations, assessing immune population interaction with tumours by imaging, and assessing the impact of the altered immune cells on tumour. These would require very significant additional experimental work and modifications to the current paper.

We have taken the reviewers' comments on board with regard to the myeloid characterisation and have reanalysed all FACS data. This includes representative dot plots at all time-points in both the MG and dLN, and we have improved the gating used based on recent publications as suggested by the reviewers (please see response to reviewer 1 above).

We agree further imaging of the interaction between leukocytes and tumour cells would be extremely interesting, especially in the context of 3D imaging. However, unfortunately (as outlined above), we are not in a position to carry out these analyses.

Reviewer 2 Comments for the Author:

Major points

The myeloid cell populations analysed are not well defined. Gr1 stains both Ly6G and Ly6C, so the emphasis on this marker is not helpful.

As above, we have now regated the myeloid cell FACS, which we hope is more comprehensive.

The original gating strategy using CD11b/Gr1 identification as a first step was influenced by Vincenzo Bronte and Dmitry Gabrilovich's historical analyses of suppressive myeloid cells. As the more recent studies by Dawson *et al.* and Jäppinen *et al.* were not available at the time these experiments were performed, our approach was to perform FACS analysis built upon strong evidence from other tissues.

We are aware that Gr1 stains Ly6C and Ly6G and is therefore in some circumstances redundant. However, the next step of the gating strategy was to subsequently look at Ly6C/Ly6G expression by CD11b+Gr1+ cells.

Use of Ly6C, Ly6G, CD11b and MHCII to define previously characterised monocyte and neutrophil populations would be better and more standard.

We have regated the myeloid FACS and provided further examples of the gating, which we hope addresses this point.

The analysis macrophage and dendritic cell populations is unclear and does not make use of current methods for their identification. Eg DCs: CD11c+MHCII+ (excluding macrophages) and separation of these into cDC1/2 using a selection of CD103/CD11b/Xcr1/CD172a.

Please see above comments. We were limited by the number of fluorophores available to us for FACS analysis and thus we did not include the suggested markers, which we agree would have been very useful. We would add that the majority of CD11c+ cells in the MG are also F4/80+ and so we are not necessarily differentiating between DCs and macrophages in this study. We agree that more markers would be beneficial for future analyses.

Macrophages should be identified with an additional marker such as CD64 or MerTK. F4/80 alone is not sufficient.

Please see above comment, we completely agree but were limited by available fluorophores.

The imaging does not add significantly to the paper and not quantified or connected to the immune populations analysed.

© 2022. Published by The Company of Biologists under the terms of the Creative Commons Attribution License (https://creativecommons.org/licenses/by/4.0/).

The purpose of the imaging in Figure 1 is to demonstrate to the reader the level of tissue remodelling/development which occurs during mammary involution. We do not expect all readers to be familiar with this tissue. Moreover, because the TUBO syngeneic model is less common than other breast cancer models e.g. 4T1, we felt it important to provide readers with the basic knowledge that these tumours are readily leukocyte-infiltrated as the epithelial tissue already has a population of intercalated leukocytes.

Immune cell distribution should be quantified to back up statements made in the text.

We agree we could have quantified leukocytes in our images. But as stated above, the images were to provide context for the reader and as we have quantified the immune cells by FACS, we do not feel it necessary to provide histological quantification.

Imaging of CD45 and SMA (F1A) during involution is already published by the authors in the FEBS journal 2020 so does not provide new information in this paper.

Please see above. We are not suggesting that these images provide new information, but we think that these images are essential to put our analysis of different involution time points in context. The remarkable removal of the majority of alveoli in the first 6 days after forced involution (by programmed cell death) is the changing environment into which tumour cells were implanted.

The immune characterisation and tumour analysis are not connected in this study as is implied by the title and abstract.

We thank the reviewer for this feedback and have rewritten the title and much of the text to reflect that our data are correlative.

Functional data for some immune subsets are needed eg. Does the change between day 3 and 6 exist in mice depleted of neutrophils by anti-Ly6G, or macrophages by anti-Csf1r? Which immune populations interact with the tumour upon implantation to implicate them in rejection?

We agree with the reviewer, that these would be very interesting experiments. However, such extensive work is beyond the scope of this manuscript.

Minor points

The lymphoid cell analysis is thorough and the observed changes in T cell subsets are interesting, but these could be analysed and presented in a more intuitive way for readers to understand. Eg. Pie charts that show global shifts over the different stages.

We have now provided pie charts of frequencies among parent populations and we agree that these enable a far more intuitive way for the reader to interact with the data.

The analysis would be clearer if individual populations were separated, then activation markers examined on these. Aligning the different organ analyses and moving the relative % measurements to the supps would aid interpretation.

Please see above. We have made every effort to simplify the immune cell characterisation so that the reader is provided with the main take-away facts for each subset. We are grateful to reviewer 2 for this suggestion.

F3: dots are too small for colours to be seen.

As above, we have simplified the myeloid gating strategy so that coloured dots are not required for data interpretation. We hope the use of pie-charts helps in this regard.

F3A Gr1- CD11b-/low looks like a single population.

We have addressed this point for reviewer 1 above; all FACS analysis now includes representative dot plots for each time-point examined which helps justify the gate positioning.

© 2022. Published by The Company of Biologists under the terms of the Creative Commons Attribution License (https://creativecommons.org/licenses/by/4.0/).

F3Q-S: low macrophage percentage does not fit with the known abundance of macrophages in the mammary gland, especially in lactation.

We agree with this comment and have now reanalysed the myeloid data using a more comprehensive gating strategy which clearly demonstrates the considerable influx of macrophages at d3Inv.

F6A-B: were use of Bl/6 vs Balb/c studs controlled for in this data?

We did not control for the breed of stud used and because the males were only used to impregnate the females, we did not feel that this was relevant; no live pups were kept beyond 22 days of birth (10d lactation + 11d involution).

We found that C57BL/6 males were generally more successful studs than Balb/c. However, all studs used were proven before they were used in involution studies.

F6B: define TLL

Total littler lost i.e. the female was visibly pregnant and was therefore individually housed ready to litter, but no pus were ever observed. Most likely the pregnancy was reabsorbed before term, or the female cannibalised the pups.

F6E-F: label young/old

Done

F6D/F, C/E: data should only be shown once

We have revised the text to clearly state where data has been replotted for comparative purposes.

F6F,I: clearly define ND, this is often used as not detected

This is defined in the legend; not determined

Line 254: define monocytic myeloid cells

We have now revised this analysis and this text is no longer included.

Lines 138: some of these references are not appropriate for preclinical cancer models during involution
Methods:
Specify the media that the tumour cells injected in. PBS. Done
Line 698: reference does not use Ficoll gradient cell enrichment This reference has been removed during editing
Specify density centrifugation speeds This is included.
Does Ficoll enrichment change population frequencies? We have not addressed this. Line 735: Define NBF Neutral Buffered Formalin. Done.

These minor points have all been addressed in the text.

In summary, we have made substantial revisions to the text to address many of the reviewers' comments and criticisms. We have carried out a complete re-analysis of all FACS data and presented this in our revised manuscript and have provided more evidence (in terms of additional representative FACS plots at all time-points) to deliver a more complete picture. As stated, we are unable to carry out any additional mouse work. We are extremely grateful for the detailed comments from the reviewers and we hope that our revisions are satisfactory. We look forward to hearing from you.

© 2022. Published by The Company of Biologists under the terms of the Creative Commons Attribution License (https://creativecommons.org/licenses/by/4.0/).

Second decision letter

MS ID#: DEVELOP/2021/200162

MS TITLE: The immune environment of the mammary gland fluctuates during post-lactational regression and correlates with tumour growth rate

AUTHORS: Christine J Watson, Jessica Hitchcock, Katherine Hughes, Sara Pensa, and Bethan Lloyd-Lewis

I have now received all the referees reports on the above manuscript, and have reached a decision. The referees' comments are appended below, or you can access them online: please go to BenchPress and click on the 'Manuscripts with Decisions' queue in the Author Area.

The overall evaluation is positive and we would like to publish a revised manuscript in Development, provided that the referees' comments can be satisfactorily addressed. Please attend to all of the reviewers' comments in your revised manuscript and detail them in your point-by-point response. If you do not agree with any of their criticisms or suggestions explain clearly why this is so.

We are aware that you may currently be unable to access the lab to undertake experimental revisions. If it would be helpful, we encourage you to contact us to discuss your revision in greater detail. Please send us a point-by-point response indicating where you are able to address concerns raised (either experimentally or by changes to the text) and where you will not be able to do so within the normal timeframe of a revision. We will then provide further guidance. Please also note that we are happy to extend revision timeframes as necessary.

Reviewer 1

Advance summary and potential significance to field

na

Comments for the author

The authors have made some improvements to their manuscript.
While they have not provided a deeper characterization of the immune landscape during lactation/involution or included immune cell analysis during tumor growth/initiation, they re-analyzed their flow cytometry data and better defined the populations.
I still have some comments on this:

- They define Ly6Clo and Ly6C- macrophages, these two should be combined as it is not a clear separation (see for example Fig. 2D).
- They mention Ly6G+ macrophages. This is very confusing as macrophages do not express Ly6G. These are surely neutrophils (Fig. 2M-N)?
- They mention (line 169) that the percentage of cells expressing F4/80 increases at d3Inv and d11Inv, yet is markedly reduced at d6Inv (Fig. 2G). This is misleading as the total cell number is clearly drastically reduced at d11Inv, as can be seen in all FACS plots for d11Inv in Figure 2. Why do they not show total cell numbers per mammary gland or g tissue for all the different cell populations to show the actual changes?
- Along these lines, they write that ‘the trend for reduced CD11b expression among F4/80+ cells during involution is not observed at d6Inv and may reflect a distinct shift in macrophage homeostasis at this time’. It is not clear what the authors mean with shift in macrophage homeostasis and reduced CD11b expression.

There are two distinct macrophage populations. The CD11b- populations increases during lactation and decreases again from d6Inv on. However, also here, Fig. 2K-L, suggests that the F4/80+CD11b+ cells expand yet this is just the percentage. As seen in Fig. 2I it is the CD11b- population that disappears. And at d11Inv there are hardly any cells left, which is also clearly evident in Fig. 2U. Total cell numbers for all these populations (as shown for CD103+ cells in Fig. 2T) should be provided.

© 2022. Published by The Company of Biologists under the terms of the Creative Commons Attribution License (https://creativecommons.org/licenses/by/4.0/).

- It is unclear why they look at CD103 expression on F4/80+ cells. If they would like to analyze DCs (which are F4/80-), a different gating strategy should be used. They show that some F4/80+CD11b-CD11c+ cells are CD103+ (Fig. S1J-k), what are these cells?
- They state that MHCII expression is significantly decreased during involution. Representative plots for MHCII should be shown.
- Ly6C+ cells (CD11b+) are monocytes (Fig.2), this should be mentioned.

Other comments:
- Have they analyzed the immune compartment in aged mice at the different post-partum and involution stages in comparison to young mice? This should be added or at least discussed.
- It should also be discussed in more detail why there could be a difference in tumor growth between d3Inv and d6Inv but not between virgin and either d3Inv or d6Inv (Fig. 7). Conversely, the biggest difference in the immune cell landscape and in particular immune cell numbers can be seen between virgin and d3Inv or d6Invs, and between d11Inv and d3Inv/c6Inv, while d11Inv resembles the virgin immune cell composition (Fig.
2). In addition to immune cells, the different tissue environments, including stromal cells, epithelial cells adipocytes, likely contribute differently to tumor formation at the different time points.

Reviewer 2

Advance summary and potential significance to field

This study presents FACS analysis of the immune environment during mouse mammary gland involution and analysis of the growth of syngeneic tumors implanted at different involution time points. Immune cell populations, particularly macrophages (although this is already known) and gtT cells are altered throughout involution. Tumor cells implanted in the mammary gland at 6 days involution grew slower than those implanted at 3 days involution. The research is interesting to the field but is undeveloped in the aspects of quantifying precise myeloid populations and experimentally connecting the immune environment with tumor growth. These limitations have been sufficiently highlighted in the text. The authors have done well to address many of my previous comments however, I still have some issues and suggestions to strengthen the paper.

Comments for the author

Major points:
Ly6C-hi and Ly6G+ cells must be excluded from the macrophage analysis. These are monocytes and neutrophils (mono/neut), which can express low levels of F4/80.
This also creates an issue in Figure 3, since many mono/neut are lost in the F4/80– gate (Fig 2M). Mono/neut should be identified using Ly6C, Ly6G and CD11b.
Monocytes and neutrophils should be discussed as their cell type as they are clearly identifiable. The claims about anti-inflammatory drugs are prominent in the abstract and discussion but require more experimental support, otherwise they should be altered to address the limitations. The difference between the transplantation routes is striking, with the difference between 3 and 6 days only being seen with non-invasive tumor cell injection. The authors state that this is due to anti-inflammatory drugs disrupting the affect of the immune environment of the tumors. However, the use of anti-inflammatory analgesics is not controlled for since the two approaches implant the cells in different ways that could also influence tumor growth. The difference could potentially be due to different sites of implantation, especially given that the exact site cannot be seen when injecting through the skin. Inflammation could also be influenced by the invasive surgery. Supplementary information showing that the implantation site is within the mammary gland, not subcutaneous, would be helpful. The authors would need to perform the surgery free cell injection both with and without analgesics to confirm that analgesics suppress the effects of inflammation on tumor growth, but I understand that this may not be possible.
Alternative causes for the difference in tumour growth between 3 days and 6 days involution should be discussed. The changing extracellular matrix (Lyons et al.,
2011, Nat Med), apidose regeneration (Zwick et al., 2018 Nat Comms) and systemic factors (Feng et al., 1995, J Cell Biol) could all affect tumor initiation.

© 2022. Published by The Company of Biologists under the terms of the Creative Commons Attribution License (https://creativecommons.org/licenses/by/4.0/).

Minor points
The immune analysis could be simplified to help readers. The analysis spans 5 figures with panels from A – S/W but is derived from 2 sets of experiments (myeloid FACS and lymphoid FACS). The figures contain some unnecessary plots making the paper more difficult to follow. Eg. For graph pairs that show %+ and %- demonstrate the same thing, one of these could be removed.
Fig1e-h and i-l: show absolute macrophage number
Make all FACS gates more bold
Fig 2b: exclude Ly6c-hi and Ly6G+ cells before analysing macrophages. These are monocytes and neutrophils
Fig 2b, f: use more contrasting colors (this also applies to these same colors in the other charts).
Fig 2D,M: virgin FACS data is shown twice. Please remove one of these
Line 181: The Ly6G+ cells are neutrophils not macrophages and should be excluded prior to F4/80
Line 173: The authors should comment on CD11b- cells potentially being distinct CD11b- cell types such as ductal macrophages, as well as the mentioned possibility of downregulation
Paragraph, line 395: as above. CD11b- macrophages in lactation/early involution are ductal macrophages and should be discussed as such.
Line 187: CD11c is not a unique DC marker as it can be expressed on macrophages
Line 271: data shows a decrease in gdT cell number but the text gives the impression they are increasing
Fig 5e: the gate has changed at 6dinv but the population spread looks the same.
Is there really a difference at 6d inv?
Fig 6a: The demonstrated gate does not select for CD8+
Line 303: FigS5 – no panel reference
Line 314-320: Figure references are out of order and confusing
Line 408: Bone-marrow derived macrophages can also be tissue resident. This is stated in the below text but the first sentence is misleading. When embryonic macrophages are removed, BM macrophages replace these with almost equivalent phenotype and function (eg. Scott et al., Nat Comms, 2016)
Line 154: Cite Dawson et al 2020 who analyse myeloid cells during involution
Line 382: Plaks et al 2015 and Jappinen et al., 2019 do not analyse involution immune cells
The recently published study by Wilson et al., 2021, Immunology, should be discussed as they analyse myeloid cell frequencies during involution

---

Second revision

Author response to reviewers' comments

Dear Florent,

We would like to thank both the editors and the reviewers for providing us with the opportunity to further modify our manuscript and appreciate very much their guidance and suggestions. We have taken on board these comments and have performed a new analysis of the FACS data. We hope that this new analysis incorporates sufficiently the reviewers' additional suggestions. The reviewer's comments are addressed individually below.
Reviewer 1 Comments for the author
The authors have made some improvements to their manuscript. While they have not provided a deeper characterization of the immune landscape during lactation/involution or included immune cell analysis during tumor growth/initiation, they re-analyzed their flow cytometry data and better defined the populations. I still have some comments on this:

-They define Ly6Clo and Ly6C- macrophages, these two should be combined as it is not a clear separation (see for example Fig. 2D).
As mentioned above, we have completely reanalysed (once again) the FACS data to incorporate these suggestions. We have now gated initially on Ly6C and Ly6G so that monocytes and neutrophils can be excluded from any macrophage analysis. We had not done this originally due to the promiscuity of Ly6C expression; Ly6C can be expressed among heterogeneous macrophage populations. However, we agree with the reviewer that prior Ly6C exclusion has enabled more

© 2022. Published by The Company of Biologists under the terms of the Creative Commons Attribution License (https://creativecommons.org/licenses/by/4.0/).

succinct analysis of the macrophages (discussed separately), and has enabled clear identification of CD11b Ly6C+ monocytes.

-They mention Ly6G+ macrophages. This is very confusing as macrophages do not express Ly6G. These are surely neutrophils (Fig. 2M-N)?
As above, we have now excluded Ly6G+ cells prior to macrophage analysis so this is no longer an issue. Neutrophils are clearly identified as Ly6G+CD11b+ (Gr1+) and we do not see Ly6G expression amongst our macrophage gating (not shown).

-They mention (line 169) that the percentage of cells expressing F4/80 increases at d3Inv and d11Inv, yet is markedly reduced at d6Inv (Fig. 2G). This is misleading as the total cell number is clearly drastically reduced at d11Inv, as can be seen in all FACS plots for d11Inv in Figure 2. Why do they not show total cell numbers per mammary gland or g tissue for all the different cell populations to show the actual changes?
We have tried to incorporate this feedback by consistently providing proportions of a given population alongside the absolute number of cells this represents. We agree that overall numbers are the determining factor but we believe that presenting percentage data alongside is extremely useful when considering more subtle changes among complex populations. Thus we have now included total cell numbers per mammary gland for all populations discussed. The reader does need to take into consideration that the mammary gland enlarges during lactation and early involution due to the milk content as well as the changing epithelium. However, we were keen to capture the entire leukocyte content of the gland (including the cells within the milk); the most accurate way to do this is to process the entire gland - milk (and cells continuously leak from involuting glands immediately upon dissection, due to the shear volume of unsuckled milk).

-Along these lines, they write that 'the trend for reduced CD11b expression among F4/80+ cells during involution is not observed at d6Inv and may reflect a distinct shift in macrophage homeostasis at this time'. It is not clear what the authors mean with shift in macrophage homeostasis and reduced CD11b expression.
There are two distinct macrophage populations. The CD11b- populations increases during lactation and decreases again from d6Inv on. However, also here, Fig. 2K-L, suggests that the F4/80+CD11b+ cells expand, yet this is just the percentage. As seen in Fig. 2I it is the CD11b- population that disappears. And at d11Inv there are hardly any cells left, which is also clearly evident in Fig. 2U. Total cell numbers for all these
populations (as shown for CD103+ cells in Fig. 2T) should be provided.

We agree our phrasing was not clear and have re-written this section, providing total cell numbers (as it now fits with the revised FACS gating). We were trying to say, as you have deciphered, that there are 2 populations of which the balance switches at d6Inv. We agree it is impossible to interpret this without total cell numbers alongside.

-It is unclear why they look at CD103 expression on F4/80+ cells. If they would like to analyze DCs (which are F4/80-), a different gating strategy should be used. They show that some F4/80+CD11b-CD11c+ cells are CD103+ (Fig. S1J-k), what are these cells?

We measured CD103 expression previously on the F4/80+ cells because we observe a distinct F4/80+CD103+ population (although we agree that the F4/80 expression is low and as we have not excluded CD11c in the gating, the CD103+ cells we described could be DCs).
In our revised gating strategy, our F4/80 gate (Fig. 3B) is fairly all-encompassing to ensure inclusion of all macrophages in this gate. However, we again describe CD103 expression among F4/80+CD11chi cells and observe a distinct CD103+ CD11b- population at all time-points (with considerable variability in virgin mice) (Fig. 3G-H)
Back-gating reveals that these cells are on the gating boundary between CD11c and F480-expressing populations (light blue in Sup. Fig.2E). Therefore, we agree (and have included the back gating for clarity) that the majority of these CD103+ cells are DCs (F4/80lo/-), as have been previously described.
However, we also clearly observe some (very few) F480+ CD103+ cells which were not previously described. We presume that this CD103 expression is enabling communication/localisation/recruitment between "macrophages" and mammary epithelial cells in much the same way as

© 2022. Published by The Company of Biologists under the terms of the Creative Commons Attribution License (https://creativecommons.org/licenses/by/4.0/).

occurs with CD103+ DCs in mucosal tissues. We would be very interested to investigate this concept further but this is not possible here.
-They state that MHCII expression is significantly decreased during involution. Representative plots for MHCII should be shown.
All MHCII quantification is provided as intensity (gMFI) and for simplicity we did not include representative plots. We have now included histograms of the CD103- macrophage populations (CD11b+ and CD11b-) shown in Fig. 3 in Fig. S2F.
-Ly6C+ cells (CD11b+) are monocytes (Fig.2), this should be mentioned.
As above, we agree and have changed our gating to clearly identify Ly6C+CD11b+ cells as monocytes (Fig. 2)

Other comments:
-Have they analyzed the immune compartment in aged mice at the different post-partum and involution stages in comparison to young mice? This should be added or at least discussed.
We have not performed these analyses and agree it would be highly insightful. We anticipate that there are differences, not least because we observe such dramatic clinical phenotypes in aged mice at d3Inv which are completely absent in young mice at the same time-point. Whether this would be visible by FACS is moot.

-It should also be discussed in more detail why there could be a difference in tumor growth between d3Inv and d6Inv but not between virgin and either d3Inv or d6Inv (Fig. 7). Conversely, the biggest difference in the immune cell landscape and in particular immune cell numbers can be seen between virgin and d3Inv or d6Invs, and between d11Inv and d3Inv/c6Inv, while d11Inv resembles the virgin immune cell composition (Fig. 2).
It is unclear why there are differences in tumour growth between d3Inv and d6Inv and not between virgin and either involution time-point. It is likely that these differences are intertwined with other physiological processes occurring at each distinct stage. It is worth noting that a high proportion of epithelial cells in the lactating gland are binucleate (essential for efficient milk production) and therefore genomically unstable. We also speculate that pregnancy provides a level of protection (be it immune-mediated or by other means e.g. hormonal or epigenetic), as observed for humans because, when using the analgesic-free/non-invasive route of tumour injection, pregnancy control mice also have a trend for slower growing tumours than virgin mice (Fig. S10H-J). However, this did not reach statistical significance.
In addition to immune cells, the different tissue environments, including stromal cells, epithelial cells, adipocytes, likely contribute differently to tumor formation at the different time points.

We have now included discussion on this and agree these factors will likely vary in their influence at different stages of involution.

Reviewer 2 Advance summary and potential significance to field
This study presents FACS analysis of the immune environment during mouse mammary gland involution and analysis of the growth of syngeneic tumors implanted at different involution time points. Immune cell populations, particularly macrophages (although this is already known) and gtT cells are altered throughout involution. Tumor cells implanted in the mammary gland at 6 days involution grew slower than those implanted at 3 days involution. The research is interesting to the field but is undeveloped in the aspects of quantifying precise myeloid populations and experimentally connecting the immune environment with tumor growth. These limitations have been sufficiently highlighted in the text. The authors have done well to address many of my previous comments, however, I still have some issues and suggestions to strengthen the paper.

Reviewer 2 Comments for the author

Major points:

Ly6C-hi and Ly6G+ cells must be excluded from the macrophage analysis. These are monocytes and neutrophils (mono/neut), which can express low levels of F4/80. This also creates an issue in Figure 3, since many mono/neut are lost in the F4/80– gate (Fig 2M). Mono/neut should be identified using Ly6C, Ly6G and CD11b. Monocytes and neutrophils should be discussed as their cell type as they are clearly identifiable.

© 2022. Published by The Company of Biologists under the terms of the Creative Commons Attribution License (https://creativecommons.org/licenses/by/4.0/).

We are grateful for such succinct feedback and we have reanalysed the myeloid FACS panel as per these comments; Ly6C+CD11b+ monocytes and Ly6G+CD11b+ neutrophils are gated among live, singlet, CD45+ cells prior to examination of any other markers. We have now discussed the dynamics of monocytes and neutrophils exclusively.

The claims about anti-inflammatory drugs are prominent in the abstract and discussion but require more experimental support, otherwise they should be altered to address the limitations. The difference between the transplantation routes is striking, with the difference between 3 and 6 days only being seen with non-invasive tumor cell injection. The authors state that this is due to anti-inflammatory drugs disrupting the affect of the immune environment of the tumors. However, the use of anti-inflammatory analgesics is not controlled for, since the two approaches implant the cells in different ways that could also influence tumor growth. The difference could potentially be due to different sites of implantation, especially given that the exact site cannot be seen when injecting through the skin. Inflammation could also be influenced by the invasive surgery. Supplementary information showing that the implantation site is within the mammary gland, not subcutaneous, would be helpful. The authors would need to perform the surgery free cell injection both with and without analgesics to confirm that analgesics suppress the effects of inflammation on tumor growth, but I understand that this may not be possible.
We have now included photographs demonstrating the intra-mammary implantation site following non-invasive tumour cell injection (Fig. S9G). Only tumours which were seeded within the fat-pad as shown were considered in our analyses.
We have not performed the controlled studies you suggest to determine whether the differing tumour growth (especially at d6Inv) is influenced by the use of analgesics and/or sedation, and we are unable to perform these studies at this point in time, but agree they would be informative. We have reworded the relevant sections to clearly state the limitations in our study.

Alternative causes for the difference in tumour growth between 3 days and 6 days involution should be discussed. The changing extracellular matrix (Lyons et al., 2011, Nat Med), apidose regeneration (Zwick et al., 2018 Nat Comms) and systemic factors (Feng et al., 1995, J Cell Biol) could all affect tumor initiation.
We have now included these alternative/additional factors in the discussion and have included some of the citations you suggest.

Minor points:

The immune analysis could be simplified to help readers. The analysis spans 5 figures with panels from A – S/W but is derived from 2 sets of experiments (myeloid FACS and lymphoid FACS). The figures contain some unnecessary plots, making the paper more difficult to follow. Eg. For graph pairs that show %+ and %- demonstrate the same thing, one of these could be removed.
We have now re-gated the myeloid FACS in a more succinct fashion to address this point and have removed the unnecessary (duplicative) plots. One exception to this is in revised Fig. 3C-D where we have kept both the %+ and %- because we go on to further dissect these populations and believe these initial histograms provide a useful visualisation of the starting point for downstream gating.

Fig1e-h and i-l: show absolute macrophage number
Whilst these exact plots no longer exist (revised gating strategy), we have now provided absolute cell numbers alongside each discussed population.

Make all FACS gates more bold
Done

Fig 2b: exclude Ly6c-hi and Ly6G+ cells before analysing macrophages. These are monocytes and neutrophils
Please see above, this is now done.

Fig 2b, f: use more contrasting colors (this also applies to these same colors in the other charts).
All pie-charts have been revised and we hope you are satisfied with the updated colours.

Fig 2D,M: virgin FACS data is shown twice. Please remove one of these
This has been done.

© 2022. Published by The Company of Biologists under the terms of the Creative Commons Attribution License (https://creativecommons.org/licenses/by/4.0/).

Line 181: The Ly6G+ cells are neutrophils not macrophages and should be excluded prior to F4/80
As above; done.

Line 173: The authors should comment on CD11b- cells potentially being distinct CD11b- cell types such as ductal macrophages, as well as the mentioned possibility of downregulation
We agree this is an important point which we have now addressed, both in the results section and the discussion. Thank you for this suggestion.

Paragraph, line 395: as above. CD11b- macrophages in lactation/early involution are ductal macrophages and should be discussed as such.
We have revised the text here.

Line 187: CD11c is not a unique DC marker as it can be expressed on macrophages
We agree with the reviewer and have in fact have not discussed DCs in any depth as our myeloid panel does not include sufficient markers to describe DCs with any certainty.

Line 271: data shows a decrease in gdT cell number but the text gives the impression they are increasing
We have now reworded much of the T cell section, taking into consideration the reviewer's advice to clearly differentiate between changes in frequency versus absolute cell counts.

Fig 5e: the gate has changed at 6dinv but the population spread looks the same. Is there really a difference at 6d inv?
We apologise, the wrong FACS plot had been included in this figure for the d6Iv time-point. We have amended the figure (now 5D); the frequency data presented in the pie-chart previously was correct. Thank you for highlighting this error.
Fig 6a: The demonstrated gate does not select for CD8+
The gate in Fig. 6a does not need to select for CD8 because CD8+ cells were already selected in Fig. 4A. However, this may not be clear and so we have updated the gating strategy cartoons to clarify this point. We have also modified the gate as it was oversized.

Line 303: FigS5 – no panel reference
Fig S5 is now Fig S8 and we have updated the text so that all elements of the figure are referred to. Thank you for pointing this out.

Line 314-320: Figure references are out of order and confusing
Thank you for highlighting this. We have amended the text accordingly.

Line 408: Bone-marrow derived macrophages can also be tissue resident. This is stated in the below text but the first sentence is misleading. When embryonic macrophages are removed, BM macrophages replace these with almost equivalent phenotype and function (eg. Scott et al., Nat Comms, 2016)
We have removed this section in the discussion as our data does not address lineage and so we felt this paragraph to be unhelpful to the reader.

Line 154: Cite Dawson et al 2020 who analyse myeloid cells during involution
Citation is now included.

Line 382: Plaks et al 2015 and Jappinen et al., 2019 do not analyse involution immune cells
We thank you for pointing this out and the text is now updated accordingly.

The recently published study by Wilson et al., 2021, Immunology, should be discussed as they analyse myeloid cell frequencies during involution
We thank the reviewers for highlighting this paper (which was not published (peer-reviewed)) when we first submitted our manuscript) as it provides a solid examination of myeloid populations throughout an extensive range of developmental time-points (especially with regard to ductal development) and includes some useful data surrounding chemokine utilisation by myeloid cells. We have now included this citation and discussed where relevant.
Once again, we thank both the editors and the reviewers for their continued investment in our manuscript and we believe our data is much stronger as a consequence of these detailed revisions.

© 2022. Published by The Company of Biologists under the terms of the Creative Commons Attribution License (https://creativecommons.org/licenses/by/4.0/).

Immune regulation and inflammation in the context of the mammary development cycle is an emerging field in its own right, not only providing insight into future therapeutic manipulation of Pregnancy Associate Breast Cancer, but also its relevance as a physiological model of a tolerogenic immune environment. We would highlight the recent flurry of publications in this area across a range of influential journals (Dawson et al. 2020, Jappinnen et al. 2019, Wilson et al. 2022). We believe our study not only supports these publications, but in addition, demonstrates the striking differences in tumour outgrowth at different stages of mammary gland involution.

Third decision letter

MS ID#: DEVELOP/2021/200162

MS TITLE: The immune environment of the mammary gland fluctuates during post-lactational regression and correlates with tumour growth rate

AUTHORS: Jessica Hitchcock, Katherine Hughes, Sara Pensa, Bethan Lloyd-Lewis, and Christine J Watson

I have now received all the referees reports on the above manuscript, and have reached a decision. The referees' comments are appended below, or you can access them online: please go to BenchPress and click on the 'Manuscripts with Decisions' queue in the Author Area.

The overall evaluation is positive and we would like to publish a revised manuscript in Development, provided that the referees' comments can be satisfactorily addressed. Please attend to all of the reviewers' comments in your revised manuscript and detail them in your point-by-point response. If you do not agree with any of their criticisms or suggestions explain clearly why this is so. If it would be helpful, you are welcome to contact us to discuss your revision in greater detail. Please send us a point-by-point response indicating your plans for addressing the referee's comments, and we will look over this and provide further guidance.

Reviewer 1

Advance summary and potential significance to field

The authors have satisfactorily addressed my comments.

Comments for the author

none

Reviewer 2

Advance summary and potential significance to field

The revised manuscript is well written and the data are clearly presented. The authors should be commended on distilling and interpreting the large amounts of data and interesting observations. They have addressed all of my comments. I only have some minor suggestions and can gladly recommend this for publication.

Comments for the author

Line 184: Many macrophages are CD11c- (most in virgin mice), so gating on CD11c+ cells as stated would exclude these. However, there are not clearly defined CD11c high and low macrophage populations in Fig 3b, so the actual analysis does not exclude many cells. I suggest

© 2022. Published by The Company of Biologists under the terms of the Creative Commons Attribution License (https://creativecommons.org/licenses/by/4.0/).

gating on all F4/80+ cells, stating that these had varying levels of CD11c and not referring to macrophages as F4/80+CD11c hi.
Line 219: The MHCII levels are very similar between the CD11b+ and CD11b– populations and is very variable. Perhaps it is generally a little higher in CD11b+ but the difference is small and probably not significant at most stages which is at odds with the text.
Fig 6 B,C: wrongly labelled as FoxP3+CD25–
Line 330: It is stated that the Central memory CD8+ cell number increases from 10d lact to 6d inv (Fig 6O), however, only the proportion (Fig 6N) increases.
Fig 8E–F: Some colors are missing from the young mice data.
Line 389: Indicate that no significant difference was found in the aged virgin vs 3d inv mice.
Line 419: Is this referring to all macrophages? As mentioned above, macrophages should only be referred to as F4/80+. Same for line 424 and 436.
Line 506 and 507: Guilliams et al., 2016, Immunity is not relevant here. It refers to marker identification of dendritic cells and macrophages.
Figure legends: As the time points are not continuous, the charts are bar graphs, not histograms.

Third revision

Author response to reviewers' comments

We are delighted that our revised manuscript was so favourably received and we hope we have addressed the remaining comments of Reviewer 2 (below) to your satisfaction.
Reviewer 1 Advance summary and potential significance to field
The authors have satisfactorily addressed my comments.

Reviewer 1 Comments for the author
none

Reviewer 2 Advance summary and potential significance to field
The revised manuscript is well written and the data are clearly presented. The authors should be commended on distilling and interpreting the large amounts of data and interesting observations. They have addressed all of my comments. I only have some minor suggestions and can gladly recommend this for publication.

Reviewer 2 Comments for the author
Line 184: Many macrophages are CD11c– (most in virgin mice), so gating on CD11c+ cells as stated would exclude these. However, there are not clearly defined CD11c high and low macrophage populations in Fig 3b, so the actual analysis does not exclude many cells. I suggest gating on all F4/80+ cells, stating that these had varying levels of CD11c and not referring to macrophages as F4/80+CD11c hi.
Thank you for this suggestion which we agree with completely. We have made the changes you suggest and now refer to macrophages simply as F4/80+ and have stated that these cells express CD11c to varying degrees.

Line 219: The MHCII levels are very similar between the CD11b+ and CD11b– populations and is very variable. Perhaps it is generally a little higher in CD11b+ but the difference is small and probably not significant at most stages, which is at odds with the text.
We have removed the sentence in question as we agree that MHCII expression among the two populations (CD11b+ and CD11b-) is similar at all stages and thus the sentence does not add anything to the interpretation. Thank you for highlighting this!

Fig 6 B,C: wrongly labelled as FoxP3+CD25–
Thank you for spotting this error. Figures are now corrected.

Line 330: It is stated that the Central memory CD8+ cell number increases from 10d lact to 6d inv (Fig 6O), however, only the proportion (Fig 6N) increases.

© 2022. Published by The Company of Biologists under the terms of the Creative Commons Attribution License (https://creativecommons.org/licenses/by/4.0/).

We have clarified the point we were trying to make, that the total number of CD8+ central memory cells is elevated between d10L and d6Inv relative to in virgin mice, rather than making a direct comparison between these two time-points. The previous text was not clear.

Fig 8E–F: Some colors are missing from the young mice data.
The colours absent in the young mice data are because the number of mice in these groups is zero, therefore cannot be represented visually in the column. We have added this explanation to the figure legend for clarification.

Line 389: Indicate that no significant difference was found in the aged virgin vs 3d inv mice.
We have added this text accordingly.

Line 419: Is this referring to all macrophages? As mentioned above, macrophages should only be referred to as F4/80+. Same for line 424 and 436.
We have updated the text accordingly referring to macrophages as F4/80+ cells.

Line 506 and 507: Guilliams et al., 2016, Immunity is not relevant here. It refers to marker identification of dendritic cells and macrophages.
We have removed the Guilliams et al. reference here as suggested.

Figure legends: As the time points are not continuous, the charts are bar graphs, not histograms.
Thank you for highlighting this mistake; all figure legends are appropriately updated.

We would like to express our sincere appreciation to both reviewers for their detailed comments and suggestions throughout the review of this manuscript. We look forward to hearing from you.

Fourth decision letter

MS ID#: DEVELOP/2021/200162

MS TITLE: The immune environment of the mammary gland fluctuates during post-lactational regression and correlates with tumour growth rate

AUTHORS: Jessica Hitchcock, Katherine Hughes, Sara Pensa, Bethan Lloyd-Lewis, and Christine J Watson
ARTICLE TYPE: Research Article

I am happy to tell you that your manuscript has been accepted for publication in Development, pending our standard ethics checks.

© 2022. Published by The Company of Biologists under the terms of the Creative Commons Attribution License (https://creativecommons.org/licenses/by/4.0/).
